# Estimation of separable direct and indirect effects in a continuous-time illness-death model

**DOI:** 10.1007/s10985-023-09601-y

**Published:** 2023-06-04

**Authors:** Marie Skov Breum, Anders Munch, Thomas A. Gerds, Torben Martinussen

**Affiliations:** Section of Biostatistics, Department of Public Health, University of Copenhagen, Copenhagen, Denmark

**Keywords:** Separable effects, Illness-death model, Survival analysis, Mediation analysis, Causal inference

## Abstract

In this article we study the effect of a baseline exposure on a terminal time-to-event outcome either directly or mediated by the illness state of a continuous-time illness-death process with baseline covariates. We propose a definition of the corresponding direct and indirect effects using the concept of separable (interventionist) effects (Robins and Richardson in Causality and psychopathology: finding the determinants of disorders and their cures, Oxford University Press, 2011; Robins et al. in arXiv:2008.06019, 2021; Stensrud et al. in J Am Stat Assoc 117:175–183, 2022). Our proposal generalizes Martinussen and Stensrud (Biometrics 79:127–139, 2023) who consider similar causal estimands for disentangling the causal treatment effects on the event of interest and competing events in the standard continuous-time competing risk model. Unlike natural direct and indirect effects (Robins and Greenland in Epidemiology 3:143–155, 1992; Pearl in Proceedings of the seventeenth conference on uncertainty in artificial intelligence, Morgan Kaufmann, 2001) which are usually defined through manipulations of the mediator independently of the exposure (so-called cross-world interventions), separable direct and indirect effects are defined through interventions on different components of the exposure that exert their effects through distinct causal mechanisms. This approach allows us to define meaningful mediation targets even though the mediating event is truncated by the terminal event. We present the conditions for identifiability, which include some arguably restrictive structural assumptions on the treatment mechanism, and discuss when such assumptions are valid. The identifying functionals are used to construct plug-in estimators for the separable direct and indirect effects. We also present multiply robust and asymptotically efficient estimators based on the efficient influence functions. We verify the theoretical properties of the estimators in a simulation study, and we demonstrate the use of the estimators using data from a Danish registry study.

## Introduction

Mediation analysis is an important tool in medical and epidemiological research for understanding the mechanisms that contribute to the overall effect of a treatment or exposure on an outcome of interest. Within the causal inference literature on mediation analysis, the target estimands of interest are often the natural (pure) direct and indirect effects (Robins and Greenland 1992; Pearl 2001), which together provide a nonparametric decomposition of the total treatment effect. A comprehensive overview of mediation analysis methods from a causal inference perspective can be found in VanderWeele (2015).

In this paper we study a continuous-time illness-death process where the potential mediator is the illness state. We are interested in the direct and indirect effect of a baseline exposure on the terminal event, adjusted for a set of pre-exposure covariates. This type of target estimand is often relevant when analysing real world data. We shall illustrate our method using a Danish registry study investigating the effects of dual antiplatelet therapy (DAPT) after myocardial infarction (MI) or stroke on mortality. DAPT is a treatment that combines aspirin and a second antiplatelet agent, which is often prescribed to MI or stroke patients to prevent blood clotting. It is well known that DAPT is associated with a lower risk of a recurrent cardiovascular event (Wallentin et al. 2009) which in turn is associated with increased mortality; this is the indirect effect of interest. At the same time DAPT has other effects that are associated with increased mortality, most notably it increases the risk of gastrointestinal bleeding (Kazi et al. 2015; Dinicolantonio et al. 2013); this is the direct effect of interest.

The conventional definition of natural direct and indirect effects is based on so-called cross-world quantities which require that we manipulate the mediator for each exposed individual to what would have occurred under non-exposure. Such quantities are not well defined in the illness-death setting since the mediator is effectively undefined when the terminal event occurs before the mediating event. This has implications for formulating the causal mediation targets of interest.

The term ‘semi-competing risks’ is often used in the literature when the outcome of interest is a non-terminal event that competes with a terminal time-to-event (Fine et al. 2001). We find that the definition of this term is unclear as discussed in Stensrud et al. (2021), and will refrain from using it in this paper. We will use the term "truncation" to describe the phenomenon when occurrence of the terminal event renders the intermediate event undefined, and the term "illness-death process" to describe the underlying data structure.

The challenges that arise when defining mediation targets for the illness-death models are similar to the well known challenges that arise when defining mediation targets for a survival outcome with a time-dependent mediator. Recent approaches in the literature redefine the target of interest beyond that of natural direct and indirect effects using randomized interventions (Zheng and van der 2017; Lin et al. 2017), path-specific effects (Vansteelandt et al. 2019) or separable effects (Didelez 2019; Aalen et al. 2020). While the setting in these papers is more general in that they allow for adjustment for time-varying covariates, they assume that the mediator process is measured at discrete time-points, and are thus not directly applicable to our setting where we allow the mediator process to change in continuous time.

Similar to Didelez (2019) and Aalen et al. (2020) we propose a definition of the direct and indirect effects using a treatment separation approach which is commonly referred to as the ‘separable effects’ approach (Stensrud et al. 2022, 2021) or ‘interventionist’ approach (Robins and Richardson 2021) to causal mediation analysis. Based on an idea by Robins and Richardson (2011) this approach considers a hypothetical treatment decomposition under which it is possible to consider manipulations of the mediator independently of the treatment given. This is done by assuming that treatment has two binary components, a ‘direct’ one which is thought to affect the terminal event directly, and an ‘indirect’ one which only affects survival through it’s effect on the intermediate event, and that the two components can be intervened upon separately. This makes it possible to define meaningful mediation targets even when the mediating event is truncated by death. The aim of this paper is to show how this approach can be applied to the continuous-time illness-death setting, and to derive estimators using semiparametric theory. In particular, the identifiability conditions and estimators we propose in this paper are an extension of Martinussen and Stensrud (2023), who consider similar causal targets and estimators in a continuous-time competing risk model.

The paper is organized as follows: In Sect. 2 we introduce the irreversible illness-death model as a stochastic process and describe the observed data structure. In Sect. 3 we formulate the targets of interest and present the identifiability conditions. In Sect. 4 we derive the efficient influence functions and establish their multiple robustness properties. We also suggest two estimators: a plug-in estimator based on the identifying functional and a one-step estimator based on using the efficient influence function as an estimating equation. We examine the performance of the estimators in a simulation study in Sect. 5. Section 6 illustrates the methods in the Danish registry data application. In Sect. 7 we provide further discussion. Proofs and additional technical details are given in the Appendices.

## Setting and notation

### Illness-death model

**Fig. 1:**
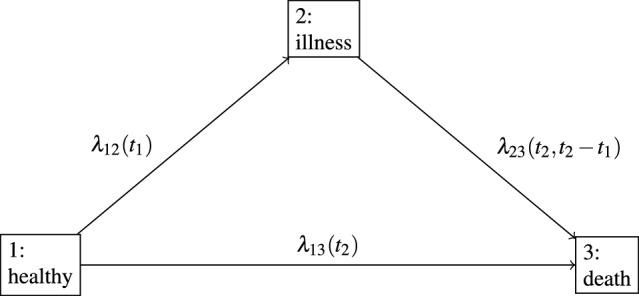
Illness-death model without recovery

We consider an irreversible illness-death model, as depicted in Fig. 1. Following Andersen et al. (2012) the illness-death model is a stochastic process $\{X(t)\}_{t \in [0, \infty )}$ with right-continuous sample paths and state space $\{1, 2, 3\}$, where state 1 is the initial ‘healthy’ state, state 2 is the intermediate ‘illness’ state and state 3 corresponds to the absorbing state ‘death’. We assume that $X(0)=1$, i.e. all subjects start in the initial ‘healthy’ state. We further assume that $2\rightarrow 1$ transitions are not possible, i.e. the process is irreversible. In our DAPT example a patient enters state 1 when experiencing a myocardial infarction (MI) for the first time. The patient stays in state 1 until they either die or experience a recurrent cardiovascular event. In the latter case the patient moves to state 2 where they remain until death.

We define time until the subject leaves state 1 $T_1 = \inf _{t>0}\{X(t) \ne 1\}$ and time until death $T_2 = \inf _{t>0}\{X(t)=3\}$. In addition to $T_1$ and $T_2$ we define the indicator

$$\begin{aligned} \eta = {\left\{ \begin{array}{ll} 1 \ \ \text { if } T_1 < T_2\\ 0 \ \ \text { if } T_1 = T_2 \end{array}\right. }. \end{aligned}$$

That is, $\eta =1$ corresponds to $X(T_1)= 2$ and $X(T_2)=3$, and $\eta =0$ corresponds to $X(T_1)=X(T_2)=3$.

Then the hazards for the transitions between states $1 \rightarrow 2$, $1 \rightarrow 3$ and $2 \rightarrow 3$, respectively, are defined as follows

$$\begin{aligned} \lambda _{12}(t)&= \lim _{\mathop {}\!\textrm{d}t \rightarrow 0} \frac{Pr(T_1 \le t+\mathop {}\!\textrm{d}t , \eta =1 \mid T_1> t)}{\mathop {}\!\textrm{d}t}, \\ \lambda _{13}(t)&= \lim _{\mathop {}\!\textrm{d}t \rightarrow 0}\frac{Pr(T_1 \le t + \mathop {}\!\textrm{d}t, \eta =0 \mid T_1> t)}{\mathop {}\!\textrm{d}t}, \\ \lambda _{23}(t, t- r)&= \lim _{\mathop {}\!\textrm{d}t \rightarrow 0} I(r \le t) \frac{Pr(T_2 \le t + \mathop {}\!\textrm{d}t \mid T_2>t, T_1=r)}{\mathop {}\!\textrm{d}t}. \end{aligned}$$

### Data structure

Let $A \in \{0,1\}$ be a baseline treatment indicator, and $W \in {\mathcal {W}}={\mathbb {R}}^d$ a vector of baseline covariates. The full uncensored data are $Z = \{T_2, T_1, \eta , A, W \} \sim Q$ where *Q* is a probability distribution belonging to a non-parametric statistical model ${\mathcal {Q}}$. Let $\mu$ be the density of *W* and $\pi (\cdot \mid W)$ be the conditional distribution of *A* given *W* which we will refer to as the propensity score. The underlying density *q* of the data *Z* under *Q* factorizes as follows

1 $$\begin{aligned} \begin{aligned} q(t, r, \eta , a, w) =&\left\{ \lambda _{12}(r \mid a, w) \lambda _{23}(t, t-r \mid a, w) S_{2}(t- \mid r, a, w) \right\} ^{\eta } \left\{ \lambda _{13}(r \mid a, w) \right\} ^{1-\eta } \\&\times S_1(r- \mid a, w) \pi (a \mid w) \mu (w), \end{aligned} \end{aligned}$$

where

$$\begin{aligned}{} & {} S_1(t \mid a, w) = \exp \left\{ -\Lambda _{12}(t \mid a, w)-\Lambda _{13}(t \mid a, w) \right\} ,\\{} & {} S_{2}(t\mid r, a, w) =\exp \left\{ - \Lambda _{23}(t, t-r \mid a, w)\right\} , \end{aligned}$$

for

$$\begin{aligned}&\Lambda _{12}(t \mid a, w) = \int _0^t \lambda _{12}(s \mid a, w) \mathop {}\!\textrm{d}s, \ \ \Lambda _{13}(t \mid a, w) = \int _0^t \lambda _{13}(s \mid a, w) \mathop {}\!\textrm{d}s, \\&\Lambda _{23}(t, t-r \mid a, w) = \int _r^t \lambda _{23}(s, s-r \mid a, w) \mathop {}\!\textrm{d}s. \end{aligned}$$

That is, $S_1$ is the survival probability for the patients in state 1 and $S_2$ is the survival probability for patients in state 2.

We also let $N_{13}(s) =I(T_2 \le s, \eta =0)$, $N_{12}(s) =I(T_1 \le s, \eta =1)$ and $N_{23}(s) = I(T_2 \le s, \eta =1)$ denote the full-data counting processes corresponding to the transitions between states $1 \rightarrow 3$, $1 \rightarrow 2$ and $2 \rightarrow 3$, respectively. In our DAPT example $N_{13}$ is the counting process which jumps when a patient in the study dies without having a recurrent cardiovascular event. Further $N_{12}$ jumps when a patient experiences a recurrent cardiovascular event, and $N_{23}$ jumps when when a patient in the study dies having experienced a recurrent cardiovascular event.

### Right censoring

We allow for right censoring with ${\tilde{C}}$ denoting the censoring variable corresponding to the time that an individual would be lost to followup. Under right censoring we only observe ${\tilde{T}}_1=T_1 \wedge {\tilde{C}}$, ${\tilde{T}}_2=T_2 \wedge {\tilde{C}}$ and the indicators $\delta = I(T_2 < {\tilde{C}})$ and ${\tilde{\eta }} = I({\tilde{T}}_1 < {\tilde{T}}_2)$. The observed data may then be represented as $O = \{{\tilde{T}}_2, \delta , {\tilde{T}}_1, {\tilde{\eta }}, A, W \} \sim P$ where *P* belongs to a non-parametric statistical model ${\mathcal {P}}$.

We may also define the observed-data counting processes $\tilde{N}_{13}(s) =I(\tilde{T_2} \le s, {{\tilde{\eta }}}=0, \delta = 1)$, ${{\tilde{N}}}_{12}(s) =I({{\tilde{T}}}_1 \le s, {{\tilde{\eta }}}=1)$ and $\tilde{N}_{23}(s) = I({{\tilde{T}}}_2 \le s, {{\tilde{\eta }}}=1, \delta =1)$ corresponding to the observed transitions between states $1 \rightarrow 3$, $1 \rightarrow 2$ and $2 \rightarrow 3$, respectively.

We make the coarsening at random (CAR) assumption, i.e., we assume that the coarsening probabilities only depend on the data as a function of the observed data. This assumption is stated more formally in Appendix A. Under CAR we can define the increments of the censoring martingale

$$\begin{aligned} \mathop {}\!\textrm{d}M_{{{\tilde{C}}}}\{u,O\}= \mathop {}\!\textrm{d}N_{{{\tilde{C}}}}(u)- \lambda _{\tilde{C}}\left\{ s;O\right\} I({{\tilde{C}}}>s) \mathop {}\!\textrm{d}s, \end{aligned}$$

where

$$\begin{aligned} \mathop {}\!\textrm{d}N_{{\tilde{C}}}(s)= I(s \le {\tilde{C}}<s+\mathop {}\!\textrm{d}s, T_1 > {\tilde{C}}) + I(s \le {\tilde{C}}< s + {\text{d}}s, T_1 < {\tilde{C}} \le T_2) \end{aligned}$$

is the censoring counting process corresponding to the observed censored observations up to and including time *s*, and

$$\begin{aligned} \lambda _{{\tilde{C}}}\left\{ s; O\right\} I({{\tilde{C}}}> s) = I({\tilde{T}}_1 > s)\alpha _{{\tilde{C}},1}(s \mid A, W) + I(T_1 \le s < {\tilde{T}}_2)\alpha _{{\tilde{C}},2}(s \mid T_1, A, W) \end{aligned}$$

is the corresponding censoring intensity. We also define

$$\begin{aligned}&K_{{{\tilde{C}}}, 1}(u \mid a, w) = \exp \left\{ -\int _0^u \alpha _{{{\tilde{C}}}, 1}(s \mid a, w) \mathop {}\!\textrm{d}s\right\} , \\&K_{{{\tilde{C}}}, 2}(v \mid u, a, w) = \exp \left\{ -\int _u^v \alpha _{{{\tilde{C}}}, 2}(s \mid u, a, w) \mathop {}\!\textrm{d}s\right\} , \end{aligned}$$

which are the probabilities of being uncensored for patients in state 1 and state 2, respectively.

## Separable direct and indirect effects

**Fig. 2:**
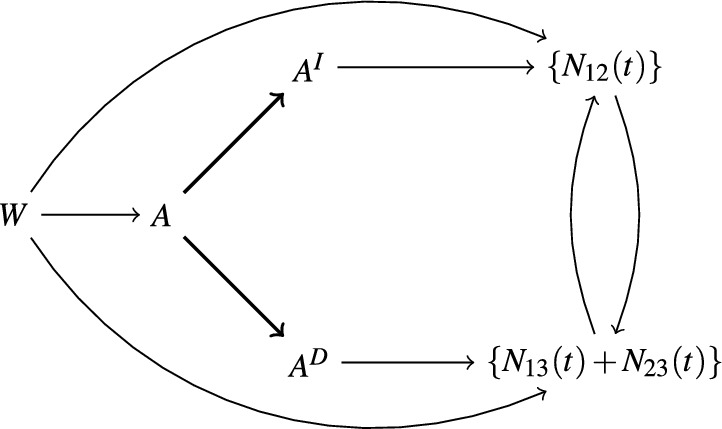
An informal causal diagram illustrating the relationship between the treatment components and the counting processes. The thick edges indicate a deterministic relationship

To define our estimand of interest we will use the concept of separable effects (Robins and Richardson 2011; Robins et al. 2021; Stensrud et al. 2022), which was briefly introduced in Sect. 1. This approach to mediation analysis moves the focus from intervening on the mediator process, which is conceptually problematic in the illness-death setting, to interventions on different components of the treatment *A*. To make the treatment decomposition more explicit we will think of the treatment *A* as having two binary components which we will denote $A^I$ and $A^D$. As depicted in Fig. 2 we will assume that the component $A^I$ only affects the terminal event through it’s effect on the intermediate event, and that the component $A^D$ only affects the terminal event directly. We will think of the corresponding four-arm trial as our ‘target trial’ and will define our target parameters based on the counterfactual variables defined by this target trial. In the observed data we have either $A^D=A^I=1$ or $A^D=A^I=0$, but we presume that an intervention is possible where $A^D \ne A^I$, i.e. the components could be set to different values. If such treatment components are assumed to exist and appropriate identification assumptions hold, then it is not necessary to conduct the four arm target trial. In fact the target parameters may be identified from the observed two-arm trial under the assumptions stated in Lemma 1 below.

This way of thinking about mediation analysis in terms of ‘separable effects’ can be useful when investigators want to know whether a specific mechanism of exposure is associated with the outcome. Often the hypothesis of interest concerns a specific ‘active ingredient’ of the exposure which may be difficult or impossible to measure.

In our example from Sect. 1 DAPT has been shown to have a protective effect on recurrent cardiovascular events, and is therefore often prescribed to MI or stoke patients. However DAPT is also associated with an increased risk of major bleeding (Wallentin et al. 2009). One of the primary forms of bleeding is gastrointestinal bleeding due to ulcers (Kazi et al. 2015; Dinicolantonio et al. 2013). We can then imagine a hypothetical treatment component $A^D$ which has the same effect as DAPT on mortality, but lacks any effect on cardiovascular events, and a hypothetical treatment component $A^I$ which has the same effect as DAPT on cardiovascular events but no direct effect on mortality. These treatment components do not necessarily correspond to meaningful real-world quantities. However, it can sometimes be useful to imagine them as hypothetical combination treatments. Assuming that gastrointestinal bleeding is the main effect of DAPT besides it’s effect on cardiovascular outcomes, the $A^I$ component would correspond to a modified treatment that does not promote ulcers. In practice, a drug that combines DAPT with an additional drug that promotes healing of ulcers and thereby nullifies the harmful effect DAPT may resemble this hypothetical treatment. For instance a recent Danish registry study has shown that proton pump inhibitors (PPI’s) can induce ulcer healing among patients treated with DAPT (Sehested et al. 2019).

It is important to note that the validity of the approach does not depend on whether the treatment components correspond to meaningful real life quantities. The validity of the approach does however depend crucially on the assumption that the two treatment components can be manipulated separately which is a strong assumption.

### Parameter of interest

For $j=1,2$ we let $T_j^{a^D,a^I}$ denote the counterfactual event times under an intervention that sets $A^D$ to $a^D$ and $A^I$ to $a^I$ and let $T_j^a$ denote the counterfactual event times under an intervention that sets $A=a$ in the observed two-arm trial.

Then, the separable direct effect (SDE) and separable indirect effect (SIE) of the illness-death model are respectively defined as

2 $$\begin{aligned} SDE(\tau , a^I)= E\left\{ I(T_2^{1,a^I}\le \tau )\right\} -E\left\{ I(T_2^{0,a^I}\le \tau )\right\} \text { for } a^I \in \{0,1\}, \end{aligned}$$

and

3 $$\begin{aligned} SIE(\tau , a^{D})= E\left\{ I(T_2^{a^D,1}\le \tau )\right\} -E\left\{ I(T_2^{a^D,0}\le \tau )\right\} \text { for } a^D \in \{0,1\}. \end{aligned}$$

where $E(\cdot )$ denotes expectations computed under the data-generating distribution.

That is, the SDE is the counterfactual contrast under $A^D=1$ and $A^D=0$ when $A^I$ is fixed at some level $a^I$. The SIE is the counterfactual contrast under $A^I=1$ and $A^I=0$ when $A^D$ is fixed at $a^D$.

Note that the separable direct and indirect effect add up to the total treatment effect

4 $$\begin{aligned} TE(\tau , a)= & {} SDE(\tau , a) + SIE(\tau , 1-a) = E\left\{ I(T_2^{1,1}\le \tau )\right\} \nonumber \\{} & {} -E\left\{ I(T_2^{0,0}\le \tau )\right\} \text { for } a \in \{0,1\}. \end{aligned}$$

### Identifiability conditions

In order to identify the parameters of the target trial given in Eqs. (2)–(3) from the observed two-arm trial we need the following assumptions

#### Lemma 1

(Identifiability) Suppose the following assumptions hold

A.0: We assume that the interventions are such that
  $$\begin{aligned} T_j^{A^D=a, A^I=a}=T_j^a \text { for } j=1,2 \end{aligned}$$
A.1: Conditional exchangeability:
A.2: Consistency: If an individual is observed to receive treatment $A=a$, then
  $$\begin{aligned} T_j^a= T_j \text { for } j=1,2 \end{aligned}$$
A.3: Positivity:
  $$\begin{aligned} \mu (w)>0 \Rightarrow \pi (a\mid W=w) > 0 \text { for } a \in \{0,1\} \text { and } w \in {\mathcal {W}}, \end{aligned}$$
  and
  $$\begin{aligned} P&(T_1>t \mid W=w)> 0 \Rightarrow \\&P({\tilde{T}}_1>t, A=a \mid W=w)>0\text { for } a \in \{0,1\}, t < \tau \text { and } w \in {\mathcal {W}}, \end{aligned}$$
  and
  $$\begin{aligned} P&(T_2>t>T_1, T_1=r \mid W=w)> 0 \Rightarrow \\&P({\tilde{T}}_2>t>T_1, T_1=r, A=a \mid W=w)>0\text { for } a \in \{0,1\}, r<t < \tau \text { and } w \in {\mathcal {W}}. \end{aligned}$$
A.4: Dismissible components conditions: for all $t \in {\mathbb {R}}, r \in {\mathbb {R}}$
  $$\begin{aligned} \lambda _{12}^{a^D=1, a^I}(t \mid W=w)&= \lambda _{12}^{a^D=0, a^I}(t\mid W=w) \text { for } a^I \in \{0,1\},\quad \quad \quad \quad \quad \quad {\Delta 1} \\ \lambda _{13}^{a^D, a^I=1}(t\mid W=w)&= \lambda _{13}^{a^D, a^I=0}(t \mid W=w) \text { for } a^D \in \{0,1\},\quad \quad \quad \quad \quad \quad {\Delta 2}\\ \lambda _{23}^{a^D, a^I=1}(t, t-r \mid W=w)&= \lambda _{23}^{a^D, a^I=0}(t, t-r\mid W=w) \text { for } a^D \in \{0,1\}.\quad \quad \quad \quad \quad \quad {\Delta 3} \end{aligned}$$
  where $\lambda _{ij}^{a^D, a^I}(\cdot )$ denotes the transition hazards of the counterfactual illness-death process under an intervention that sets $A^D=a^D$ and $A^I=a^D$.

Under assumptions A.1–A.4 we have,

5 $$\begin{aligned} E\left\{ I(T_2^{a^D,a^I}\le \tau )\right\}&= \psi (P; \tau , a^D, a^I) :=E \Bigg \{P_{13}(\tau , a^D, a^I, W)\Bigg \}, \end{aligned}$$

where

$$\begin{aligned} P_{13}(\tau , a^D, a^I, W)=&1- \Omega _{\tau }(a^D, a^I, w) -\int _{0}^{\tau } S_2(\tau |r,a^D, W) \Omega _r(a^D, a^I, w) \mathop {}\!\textrm{d}\Lambda _{12}(r|a^I,W), \end{aligned}$$

for

$$\begin{aligned} \Omega _r(a^D, a^I, w) = \exp \left\{ -\Lambda _{12}(r \mid a^I, w) - \Lambda _{13}(r \mid a^D, w)\right\} . \end{aligned}$$

Consequently, the separable direct and indirect effects are identified to

6 $$\begin{aligned} SDE(\tau , a^I) = \psi (P; \tau , 1,a^I) - \psi (P; \tau , 0, a^I), \end{aligned}$$

and

7 $$\begin{aligned} SIE(\tau , a^D) = \psi (P; \tau , a^D, 1) - \psi (P; \tau , a^D, 0). \end{aligned}$$

#### Proof

In Appendix B. $$\square $$

Assumption A.0 is a separable effects analog of the consistency assumption. Assumption A.1–A.3 are standard assumptions for causal inference. Assumption A.4 is the so-called dismissible components conditions, which is an extension of the dismissible components conditions in Martinussen and Stensrud (2023) to the illness-death setting. In particular, assumption ($\Delta 1$) states that the counterfactual hazards of the $1 \rightarrow 2$ transition are equal under all values of $a^D$, and assumption ($\Delta 2$) states that the counterfactual hazards of the $1 \rightarrow 3$ transition are equal under all values of $a^I$. Lastly assumption ($\Delta 3$) states that the counterfactual hazards of the $2 \rightarrow 3$ transition are equal under all values of $a^I$. When the treatment components correspond to meaningful real-world treatments, the dismissible components conditions are empirically verifiable in future trials.

The dismissible components conditions are violated if the $A^D$ and $A^I$ components cannot be manipulated separately. In our DAPT example this would be the case if the biological pathways through which the medication affects MI or stroke is intertwined with the pathways through which it affects bleeding. The dismissible components conditions are also violated if there is an unmeasured common cause of the risk the intermediate and the terminal event. This is similar to the classical ‘no unmeasured mediator-outcome confounding’ assumption which is needed to identify natural (in-)direct effects. In our DAPT example this would be the case if there is an unmeasured common cause of cardiovascular events such as MI or stroke, and death.

## Estimation

In this section we address the question of how to construct estimators of the estimand in Eq. (5). Efficient influence functions (EIFs) are an important concept in statistical theory for constructing estimators of causal parameters with desirable properties. In particular estimators based on the EIF are locally efficient (Bickel et al. 1993). Moreover they often exhibit multiple robustness properties in the sense that consistency of the estimator is preserved under misspecification of one or more components of the data distribution. Further, they are compatible with data adaptive estimation of nuisance parameters provided certain rate conditions hold.

In this paper we focus on the first two properties and assume (semi-)parametric models for the nuisance parameters. In particular, in what follows, we let ${\hat{\Lambda }}_{12,n}$, ${\hat{\Lambda }}_{13,n}$, ${\hat{\Lambda }}_{23,n}$, ${\hat{\Lambda }}_{{{\tilde{C}}}, n}$, ${\hat{\pi }}_n$ denote (semi-)parametric estimators for the relevant components of the data distribution, and we let $\Lambda _{12}^*$, $\Lambda _{13}^*$, $\Lambda _{23}^*$, $\Lambda _{{{\tilde{C}}}}^*$, $\pi ^*$ and denote the large sample limits in probability of the (possibly misspecified) estimators. We let $Q^*$ and $P^*$ denote the corresponding distributions of *Z* and *O* respectively. If our working model for $\Lambda _{12}$ is correctly specified then $\Lambda _{12}^*=\Lambda _{12}$ and the same holds for $\Lambda _{13}$, $\Lambda _{23}$, $\Lambda _{{{\tilde{C}}}}$ and $\pi$.

In Sect. 4.1 we derive the efficient influence function. In Sect. 4.2 we propose two types of estimators. The first is a ‘plug-in’ type estimator constructed by substituting estimators for the relevant part of the data distribution directly into (5). The second is a multiply robust estimator which uses the efficient influence function as an estimating equation. In Sect. 4.3 we provide details on how to construct estimators of their asymptotic variance.

### Efficient influence function

Below we derive the EIF of the separable direct and indirect effects under a nonparametric model. We first derive the full-data efficient influence function and then, assuming CAR and Assumptions A.0–A.4 hold, map it to the observed data efficient influence function using results given in Tsiatis (2006). We also establish general multiple robustness properties that will be satisfied by any estimator which solves the EIF estimating function.

*Full-data efficient influence function*

Let $\psi :{\mathcal {Q}}\rightarrow {\mathbb {R}}$, where $Q^* \rightarrow \psi (Q^*; \tau , a^D, a^I)=E^*\left\{ I(T_2^{a^D,a^I}\le \tau )\right\}$ and $E^*(\cdot )$ denotes the expectation computed under $Q^*$. In Appendix C we show that the efficient influence function for $\psi$ at $Q^*$ is given by

8 $$\begin{aligned} \begin{aligned} {\tilde{\psi }}(Q^*)(Z; \tau , a^D, a^I) =&\frac{I(A=a^{I})}{\pi ^*(a^{I}\mid W)} \int _0^{\tau } \frac{h_{12,\tau }^*(s, a^D, a^I, W)\mathop {}\!\textrm{d}M_{12}^{F*}(s, a^I, W)}{S_1^*(s \mid a^I, W)} \\&+ \frac{I(A=a^D)}{\pi ^*(a^D \mid W)} \Bigg \{\int _0^{\tau } \frac{ h_{13,\tau }^*(s, a^D, a^I, W) \mathop {}\!\textrm{d}M_{13}^{F*}(s, a^D, W)}{S_1^*(s \mid a^D, W)} \\&+ \frac{\eta h_{23,\tau }^*( T_1, a^D, a^I, W)}{S_1^*(T_1 \mid a^D, W)} \int _{T_1}^{\tau } \frac{\mathop {}\!\textrm{d}M_{23}^{F*}(s,T_1, a^D, W)}{S_2^*(s \mid T_1, a^D, W)} \Bigg \} \\&+ P_{13}^*(\tau , a^D, a^{I}, W) - \psi (Q^*; \tau , a^D, a^I), \end{aligned} \end{aligned}$$

with

$$\begin{aligned} h_{1j,\tau }^*(s, a^D, a^I, w) =&\Omega ^*_{\tau }(a^D, a^I, w) + \int _s^{\tau } S_2^*(\tau \mid r, a^D, w) \Omega _r^*(a^D, a^I, w)\mathop {}\!\textrm{d}\Lambda _{12}^*(r\mid a^I, w) \\ {}&+ {\left\{ \begin{array}{ll}- S_2^*(\tau \mid s, a^D, w) \Omega _s^*(a^D, a^I, w), &{}\text {when } j=2 \\ 0, &{}\text {when } j=3 \end{array}\right. }, \end{aligned}$$

and

$$\begin{aligned} h_{23,\tau }^*(s, a^D, a^I, w) =&\frac{\lambda _{12}^*(s \mid a^I, w)}{\lambda _{12}^*(s \mid a^D, w)} \Omega _{s}^*(a^D, a^I, w)S_2^*(\tau \mid s, a^D, w), \end{aligned}$$

and where $\mathop {}\!\textrm{d}M_{ij}^{F*}$ denote the full-data martingale increments under $Q^*$

$$\begin{aligned} \mathop {}\!\textrm{d}M_{13}^{F*}(s, A, W)&= \mathop {}\!\textrm{d}N_{13}(s)-\lambda _{13}^*(s \mid A, W)I(T_1>s)\mathop {}\!\textrm{d}s,\\ \mathop {}\!\textrm{d}M_{12}^{F*}(s, A, W)&= \mathop {}\!\textrm{d}N_{12}(s) - \lambda _{12}^*(s \mid A, W) I(T_1>s) \mathop {}\!\textrm{d}s,\\ \mathop {}\!\textrm{d}M_{23}^{F*}(s, T_1, A, W)&= \mathop {}\!\textrm{d}N_{23}(s) - \lambda _{23}^*(s, s-T_1\mid A, W) I(T_1 \le s < T_2) \mathop {}\!\textrm{d}s. \end{aligned}$$

#### Lemma 2

(Multiple robustness) The full-data efficient influence function admits a multiple robust structure in the sense that $E \left\{ {\tilde{\psi }}(Q^*)(Z) \right\} = \psi (Q) - \psi (Q^*)$ if one of the following holds

(i) $\pi ^*(a \mid w)=\pi (a \mid w), \Lambda _{12}^*(r \mid a, w)=\Lambda _{12}(r \mid a, w)$ and $\Lambda _{23}^*(t, t-r \mid a, w)=\Lambda _{23}(t, t-r \mid a, w)$ for all $t,r \in [0, \tau ]$, $a \in \{0,1\}$ and almost all *w*,
(ii) $\pi ^*(a \mid w)=\pi (a \mid w), \Lambda _{13}^*(t \mid a, w)= \Lambda _{13}(t \mid a, w)$ and $\Lambda _{23}^*(t, t-r \mid a, w)=\Lambda _{23}(t, t-r \mid a, w)$ for all $t,r \in [0, \tau ]$, $a \in \{0,1\}$ and almost all *w*,
(iii) $\pi ^*(a \mid w)=\pi (a\mid w), \Lambda _{12}^*(t \mid a, w)= \Lambda _{12}(t \mid a, w)$ and $\Lambda _{13}^*(t \mid a, w)=\Lambda _{13}(t \mid a, w)$ for all $t \in [0, \tau ]$, $a \in \{0,1\}$ and almost all *w*.

#### Proof

In Appendix E. $$\square $$

The multiple robustness properties stated in the lemma above imply that the full-data influence function ${\tilde{\psi }}(Q^*)(Z)$ is a consistent estimating function of $\psi (Q)$ when at most one of the transition intensities is inconsistently estimated.

*Observed-data efficient influence function* Let $\psi :{\mathcal {P}}\rightarrow {\mathbb {R}}$, where $P^* \rightarrow \psi (P^*; \tau , a^D, a^I)=E^*\left\{ I(T_2^{a^D,a^I}\le \tau )\right\}$. In Appendix D we show that the observed data efficient influence function is given by

9 $$\begin{aligned} \begin{aligned}&{\tilde{\psi }}(P^*)(O; \tau , a^D, a^I) \\&\quad = \frac{I(A=a^I)}{\pi ^*(a^I\mid W)} \int _0^{\tau } \frac{h_{12,\tau }^*(s, a^D, a^I, W)}{K^*_{{\tilde{C}},1}(s \mid a^I, W)}\frac{\mathop {}\!\textrm{d}M_{12}^*(s, a^I, W)}{S_1^*(s \mid a^I, W)} \\&\qquad + \frac{I(A=a^D)}{\pi ^*(a^D \mid W)} \Bigg \{\int _0^{\tau } \frac{h_{13,\tau }^*(s, a^D, a^I, W)}{K_{{\tilde{C}},1}^*(s \mid a^D, W)} \frac{\mathop {}\!\textrm{d}M_{13}^*(s, a^D, W)}{S_1^*(s \mid a^D, W)} \\&\qquad + \frac{{\tilde{\eta }}h_{23,\tau }^*({\tilde{T}}_1, a^D, a^I, W)}{S_1^*({\tilde{T}}_1 \mid a^D, W)K^*_{{\tilde{C}}, 1}({\tilde{T}}_1 \mid a^D, W)} \int _{{\tilde{T}}_1}^{\tau } \frac{\mathop {}\!\textrm{d}M_{23}^*(s,{\tilde{T}}_1, a^D, W)}{S_2^*(s \mid {\tilde{T}}_1, a^D, W)K_{{\tilde{C}}, 2}^*(s \mid {\tilde{T}}_1, a^D, W)} \Bigg \} \\&\qquad + P_{13}^*(t, a^D, a^I, W) - \psi (P^*; t, a^D, a^I), \end{aligned} \end{aligned}$$

with $\mathop {}\!\textrm{d}M_{ij}^*(\cdot )$ denoting the observed-data martingale increments under $P^*$

$$\begin{aligned} \mathop {}\!\textrm{d}M_{12}^*(s, A, W)&= \mathop {}\!\textrm{d}{{\tilde{N}}}_{12}(s) - \lambda ^*_{12}(s \mid A, W) I({\tilde{T}}_1>s) \mathop {}\!\textrm{d}s,\\ \mathop {}\!\textrm{d}M_{13}^*(s, A, W)&= \mathop {}\!\textrm{d}{{\tilde{N}}}_{13}(s) - \lambda ^*_{13}(s \mid A, W) I({\tilde{T}}_1>s) \mathop {}\!\textrm{d}s,\\ \mathop {}\!\textrm{d}M_{23}^*(s, {\tilde{T}}_1, A, W)&= \mathop {}\!\textrm{d}{{\tilde{N}}}_{23}(s) - \lambda _{23}^*(s, s-{\tilde{T}}_1 \mid A, W) I({\tilde{T}}_1 \le s <{\tilde{T}}_2) \mathop {}\!\textrm{d}s. \end{aligned}$$

#### Lemma 3

(Multiple robustness) The observed-data efficient influence function admits a multiple robust structure in the sense that $E \left\{ {\tilde{\psi }}(P^*)(O) \right\} = \psi (P) - \psi (P^*)$ if one of the following holds

(i) $\pi ^*(a \mid w)=\pi (a \mid w), \Lambda _{{{\tilde{C}}}}^*\{t \mid G_s(z)\} = \Lambda _{{{\tilde{C}}}}\{t \mid G_s(z)\}, \Lambda _{12}^*(r \mid a, w)=\Lambda _{12}(r \mid a, w)$ and $\Lambda _{23}^*(t, t-r \mid a, w)=\Lambda _{23}(t, t-r \mid a, w)$ for all $t,r \in [0, \tau ]$, $a \in \{0,1\}$ and almost all *w*,
(ii) $\pi ^*(a \mid w)=\pi (a \mid w), \Lambda _{{{\tilde{C}}}}^*\{t \mid G_s(z)\} = \Lambda _{{{\tilde{C}}}}(t \mid G_s(z)),\Lambda _{13}^*(t \mid a, w)= \Lambda _{13}(t\mid a, w)$ and $\Lambda _{23}^*(t, t-r \mid a, w)=\Lambda _{23}(t, t-r \mid a, w)$ for all $t,r \in [0, \tau ]$, $a \in \{0,1\}$ and almost all *w*,
(iii) $\pi ^*(a \mid w)=\pi (a \mid w), \Lambda _{{{\tilde{C}}}}^*\{t \mid G_s(z)\} = \Lambda _{{{\tilde{C}}}}\{t \mid G_s(z)\},\Lambda _{12}^*(t \mid a, w)= \Lambda _{12}(t \mid a, w)$ and $\Lambda _{13}^*(t \mid a, w)=\Lambda _{13}(t \mid a, w)$ for all $t \in [0, \tau ]$, $a \in \{0,1\}$ and almost all *w*,
(iv) $\Lambda _{12}^*(r \mid a, w)=\Lambda _{12}(r \mid a, w), \Lambda _{13}^*(t \mid a, w)= \Lambda _{13}(t \mid a, w)$ and $\Lambda _{23}^*(t, t-r \mid a, w)=\Lambda _{23}(t, t-r \mid a, w)$ for all $t,r \in [0, \tau ]$, $a \in \{0,1\}$ and almost all *w*.

#### Proof

In Appendix F. $$\square $$

This means that when the censoring distribution is correctly specified the same multiple robustness properties hold as in the full-data case. The censoring model and propensity score are allowed to be misspecified when all three transition intensites are correctly specified.

*Efficient influence functions of the separable direct and indirect effects*

Consider the mappings $P^* \rightarrow \psi ^{\textrm{SDE}}(P^*; \tau , a^D, a^I)=\psi (P^*;\tau , 1, a^I)-\psi (P^*;\tau , 0, a^I)$ for $a^I \in \{0,1\}$ and $P \rightarrow \psi ^{\textrm{SIE}}(P^*; \tau , a^D, a^I)=\psi (P^*;\tau , a^D, 1)-\psi (P^*;\tau , a^D, 0)$ for $a^D \in \{0,1\}$. It follows by the functional delta method that the efficient influence functions of the separable direct and indirect effects in (6) and (7) are given by respectively

$$\begin{aligned} {\tilde{\psi }}^{\textrm{SDE}}(P^*)(O; \tau , a^I) = {\tilde{\psi }}(P^*)(O; \tau , 1, a^I)-{\tilde{\psi }}(P^*)(O; \tau , 0, a^I), \ \text { for } a^I \in \{0,1\}, \end{aligned}$$

and

$$\begin{aligned} {\tilde{\psi }}^{\textrm{SIE}}(P^*)(O; \tau , a^D) = {\tilde{\psi }}(P^*)(O; \tau , a^D, 1)-{\tilde{\psi }}(P^*)(O; \tau , a^D, 0), \ \text { for } a^D \in \{0,1\}, \end{aligned}$$

and will inherit the multiple robustness properties established in Lemma 3.

### Estimators

*Plug-in (G-computation) estimator*

A plug-in estimator estimates the relevant part of the distribution of *O*, in this case the empirical distribution of *W* and appropriate estimators ${\hat{\Lambda }}_{12,n}, {\hat{\Lambda }}_{13,n}$ and ${\hat{\Lambda }}_{23,n}$ of the transition intensities, and substitutes them in place of the unknown quantities in Eq. (5). Then one obtains the estimator

10 $$\begin{aligned} {\hat{\Psi }}^{\text {Plug-in}}_n(\tau , a^D, a^I)=n^{-1}\sum _{i=1}^n {\hat{P}}_{13}(\tau , a^D, a^I, W_i), \end{aligned}$$

where

$$\begin{aligned} {\hat{P}}_{13}(\tau , a^D, a^I, W)&= P_{13}(\tau , a^D, a^I, W; {\hat{\Lambda }}_{12,n}, {\hat{\Lambda }}_{13,n},{\hat{\Lambda }}_{23,n}). \end{aligned}$$

Equation (5) is also known as the G-computation formula (Robins 1986), and the estimator in (10) is also referred to as a G-computation estimator. Note that consistency of ${\hat{\Psi }}^{\text {Plug-in}}_n(t, a^D, a^I)$ depends on consistency of the estimators of all three transition intensities.

*One-step estimator*

As mentioned above the efficient influence function is useful for constructing multiply robust efficient estimators. One way of doing this is to use the influence function directly as an estimating equation (van der and Robins 2003). Since the EIF in equation (9) is linear in the parameter of interest, this results the estimator:

11 $$\begin{aligned} {\hat{\Psi }}^{\text {one-step}}_n(\tau , a^D, a^I)= n^{-1}\sum _{i=1}^n \varphi ({\hat{\pi }}_n,{\hat{\Lambda }}_{12,n}, {\hat{\Lambda }}_{13,n},{\hat{\Lambda }}_{23,n}, {\hat{\Lambda }}_{\tilde{C},n})(O_i; \tau , a^D, a^I), \end{aligned}$$

where

$$\begin{aligned} \varphi (P)(O; \tau , a^D, a^I)= {\tilde{\psi }}(P)(O; \tau , a^D, a^I)+ \psi (P; t, a^D, a^I) \end{aligned}$$

The estimator in (11) is multiply robust. In particular it is consistent under misspecification of (i) $\Lambda _{12}$, (ii) $\Lambda _{13}$, (iii) $\Lambda _{23}$ or (iv) $\pi$ and $\Lambda _{{{\tilde{C}}}}$ as shown in Lemma 3.

Note that we can write:

$$\begin{aligned}{} & {} \varphi ({\hat{\pi }}_n,{\hat{\Lambda }}_{12,n}, {\hat{\Lambda }}_{13,n},{\hat{\Lambda }}_{23,n}, {\hat{\Lambda }}_{\tilde{C},n})(O; \tau , a^D, a^I) \\{} & {} \quad ={\tilde{\psi }}({\hat{\pi }}_n,{\hat{\Lambda }}_{12,n}, {\hat{\Lambda }}_{13,n},{\hat{\Lambda }}_{23,n}, {\hat{\Lambda }}_{\tilde{C},n})(O; \tau , a^D, a^I)+ {\hat{\Psi }}^{\text {G-comp}}_n(\tau , a^D, a^I). \end{aligned}$$

This approach is also referred to as a so-called ‘one-step’ bias correction approach (Ibragimov and Has’minskii 1981; Pfanzagel and Wefelmeyer 1985), and we will refer to the estimator in (11) as a ‘one-step’ estimator.

### Asymptotic variance

If all nuisance models are correctly specified, then a consistent estimator of the asymptotic variance can be obtained from the variance of the influence function. However if one or more of the nuisance models are misspecified then this variance estimator is no longer consistent, and other techniques must be used.

Suppose we are willing to assume fully parametric models for all nuisance parameters. Then we can derive the asymptotic distribution of the estimators in (10) and (11) by stacking the corresponding unbiased estimating equations for the target and nuisance parameters, and applying standard estimating equation theory (Stefanski and Boos 2002). In particular, let ${\hat{\theta }}_n$ be the estimators of the parameters of interest and nuisance parameters that solves

$n^{-1}\sum _{i=1}^n m(O_i, {\hat{\theta }}_n)=0$ where $m(O, \theta )$ are the stacked estimating equations of both the parameter of interest and nuisance parameters. For the plug-in estimator in (10) this would be ${\hat{\theta }}_n=({\hat{\Psi }}^{\text {Plug-in}},{\hat{\Lambda }}_{12,n},{\hat{\Lambda }}_{13,n}, {\hat{\Lambda }}_{23,n})$ and $m(O, \theta )=(P_{13}, S_{\Lambda _{12}}, S_{\Lambda _{13}}, S_{\Lambda _{23}})$ where $S_{\Lambda _{12}}, S_{\Lambda _{13}}, S_{\Lambda _{23}}$ are appropriate estimating equations for the transition hazards. Under suitable regularity conditions (Newey and McFadden 1994; van der Vaart 2000; Tsiatis 2006), we have

$$\begin{aligned} n^{1/2}({\hat{\theta }}-\theta ^*) \rightsquigarrow N\left( 0,E\left\{ -\frac{\partial m(O, \theta ^*)}{\partial \theta ^T} \right\} ^{-1} \text {var} \left\{ m(O, \theta ^*)\right\} E\left\{ -\frac{\partial m(O, \theta ^*)}{\partial \theta ^T} \right\} ^{-1^T}\right) . \end{aligned}$$

It is then possible to derive an analytic expression for the asymptotic variance of the estimators in (10) and (11) using the sandwich variance estimator.

When the nuisance models are e.g. Cox regression models we need to take into account the variability of the baseline hazards which may be nonparametrically estimated. Then the asymptotic distribution can be derived using the functional delta method (van der Vaart 2000). This expression becomes very complicated, especially for the one-step estimator, and deriving an explicit estimator of the variance goes beyond the scope of this paper.

## Simulation study

### Simulation study 1: empirical performance

Below, we report the results from a simulation study where the aim is to compare the finite sample performance of the plug-in estimator and the one-step estimator.

The data was generated by the following simulation procedure:

- $$W \sim Uniform(0,1)$$
- $$A \mid W \sim Bernoulli(\text {expit}(-0.5+ W+\zeta W^2))$$
- $$T_1 \mid A, W \sim Exponential(\lambda _{12}+\lambda _{13})$$ with $$\lambda _{12}=0.039 \cdot \exp (\log (2) W+ A+\gamma ^{12}AW)$$ and $$\lambda _{13}=0.026 \cdot \exp (\log (2) W+0.5 A+\gamma ^{13}(1-A)W)$$
- $$\eta \mid A, W \sim Bernoulli(\lambda _1/(\lambda _1+\lambda _2))$$
- $$T_2= T_1 + \eta \cdot U $$ with $$U \sim Exponential(\lambda _{23})$$ where $$\lambda _{23}=0.052 \cdot \exp (\log (2)W+0.5 A + \gamma ^{23}(1-A)W)$$
- $${\tilde{C}} \mid W \sim Exponential(\lambda _{{\tilde{C}}})$$ with $$\lambda _{{\tilde{C}}} = 0.035 \cdot \exp (\theta W)$$

where $\text {expit}(x)=\{1+\exp (x)\}^{-1}$. Note that this corresponds to a scenario where treatment has a protective effect on both disease and death, and where the treatment effect on death is the same in diseased and disease-free subjects.

An estimator for the propensity score was constructed using a logistic regression model with main effects only. For the transition hazards we constructed estimators using a Cox regression model with main effects only and for the censoring hazard we used a Cox model with no covariate effects. The dependency of $\Lambda _{23}$ on the time of reaching state 2 was handled by delayed entry. We considered 8 different scenarios: in scenario (i) all nuisance models were correctly specified which is the case when $(\zeta ,\gamma ^{12},\gamma ^{13},\gamma ^{23},\theta )=0$, and in scenarios (ii)–(viii) we considered misspecifications of different combinations of the nuisance models by varying the values of $(\zeta ,\gamma ^{12},\gamma ^{13},\gamma ^{23},\theta )$ accordingly. Additional details on the misspecified scenarios are given in Appendix G.

**Fig. 3:**
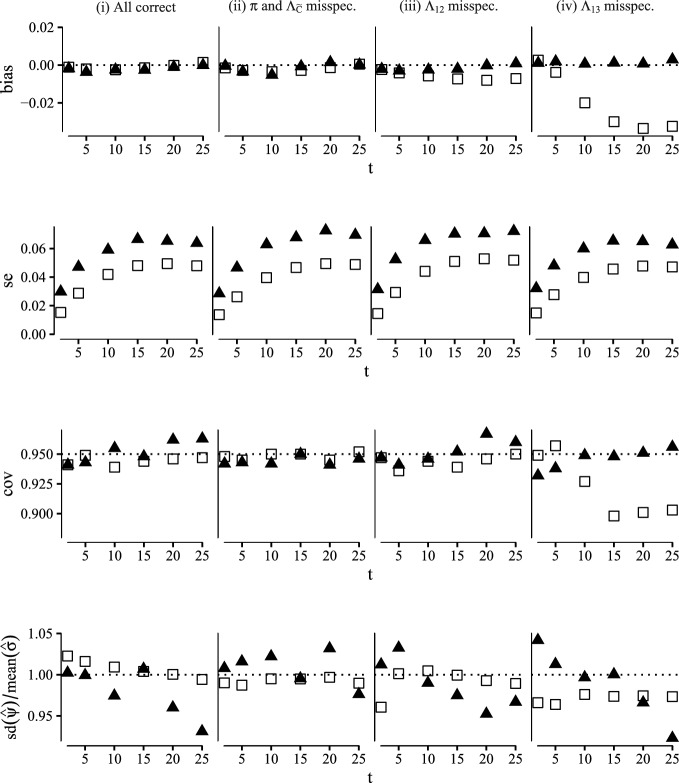
Comparison of the G-computation (white rectangles) and one-step (black triangles) estimators of the SDE computed at time points $t \in \{2, 5, 10, 15, 20, 25\}$ in terms of bias, empirical standard error, coverage of 95% confidence intervals and accuracy of the standard error estimator. This figure contains scenarios (i)–(iv) (Color figure online)

**Fig. 4:**
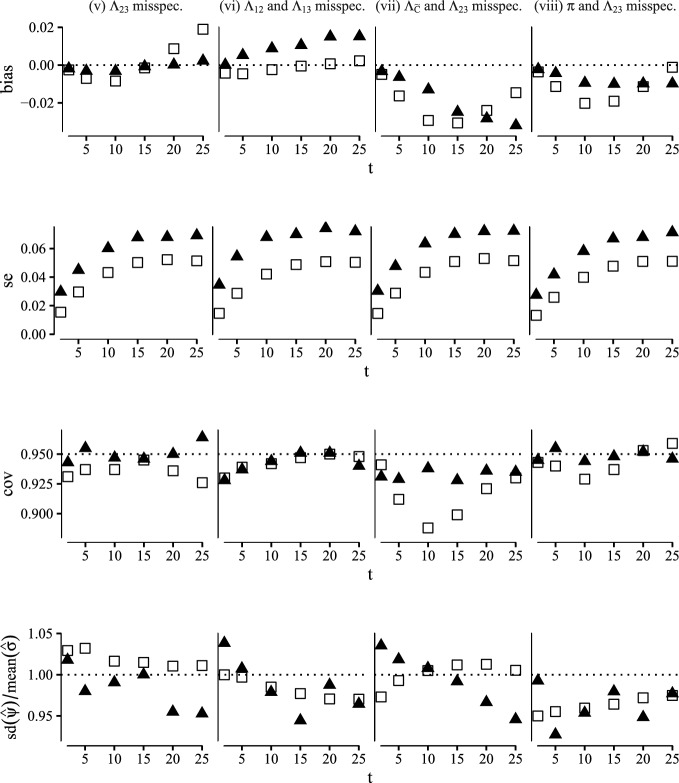
Comparison of the G-computation (white rectangles) and one-step (black triangles) estimators of the SDE computed at time points $t \in \{2, 5, 10, 15, 20, 25\}$ in terms of bias, empirical standard error coverage of 95% confidence intervals and accuracy of the standard error estimator. This figure contains scenarios (v)–(vii) (Color figure online)

For each scenario we generated 1000 datasets from the simulation procedure with a sample size of 400. For each dataset we computed the plug-in estimator and the one-step estimator for the SDE along with the bootstrap variance for each estimator based on 250 replicates. The results of our simulation study are summarized in Figs. 3 and 4 where for all scenarios we report bias, empirical standard error, coverage of the 95 % Wald confidence interval and accuracy of the standard error estimator computed at time points $t\in \{1, 5, 10, 15, 20, 25\}$.

As expected both the plug-in estimator and the one-step estimator are consistent in scenario (i) where all nuisance models are correctly specified and scenario (ii) were the propensity score and censoring models are misspecified. Moreover the coverages are close the nominal level. In scenarios (iii)–(v) where we consider misspecifications of at most one of the transition hazard models the one-step estimator provides a bias reduction over the plug-in estimator, as predicted by the multiple robustness properties in Lemma 3. In scenarios (vi)–(viii) where we consider misspecifications that go beyond the robustness properties of lemma 3 both the plug-in estimator and the one-step estimator are biased, except in scenario (vi) where the plug-in estimator surprisingly appears unbiased. The one-step estimator is more variable than the plug-in estimator throughout all scenarios.

This simulation study confirms the double robustness properties of the one-step estimator derived in Sect. 4.1, which, along with the potential compatibility with data-adaptive estimation of nuisance parameters, highlights the real-word utility of the one-step estimator.

### Simulation study 2: violation of assumptions

The dismissible components conditions in Lemma 1 are violated in the presence of an unmeasured common risk factor for illness and death. Below, we study such violations in a simulation study.

The data was generated by the following simulation procedure:

- $$W \sim Bernoulli(0.5)$$
- $$A^D \mid W \sim Bernoulli(\text {expit}(-0.5 + W))$$
- $$A^I \mid W \sim Bernoulli(\text {expit}(-0.5+ W))$$
- $$U \sim Bernoulli(0.6)$$
- $$T_1 \mid A^D, A^I, W \sim Exponential(\lambda _{12}+\lambda _{13})$$ with $$\lambda _{12}=0.039 \cdot \exp (\log (2)W+\beta ^{12}_{A} A^I + \gamma _U U)$$ and $$\lambda _{13}=0.026 \cdot \exp (\log (2)W+\beta ^{13}_{A}A^D +\gamma _U U)$$
- $$\eta \mid A^D, A^I, W \sim Bernoulli(\lambda _1/(\lambda _1+\lambda _2))$$
- $$T_2= T_1 + \eta \cdot V $$ with $$V \sim Exponential(\lambda _{23})$$ with $$\lambda _{23}=0.052 \cdot \exp (\log (2) W+\beta ^{13}_{A} A^D + \gamma _U U)$$
- $${\tilde{C}} \sim Exponential(\lambda _{{\tilde{C}}})$$ with $$\lambda _{{\tilde{C}}} = 0.035$$

We varied $\gamma _U$ along the grid $\{-1, -0.9,...,0.9,1\}$ and considered the four cases: (I) Protective treatment effect on disease and death, (II) Protective effect on disease and harmful effect on death, (III) Harmful effect on disease and protective effect on death and (IV) Harmful treatment effect on disease and death.

**Fig. 5:**
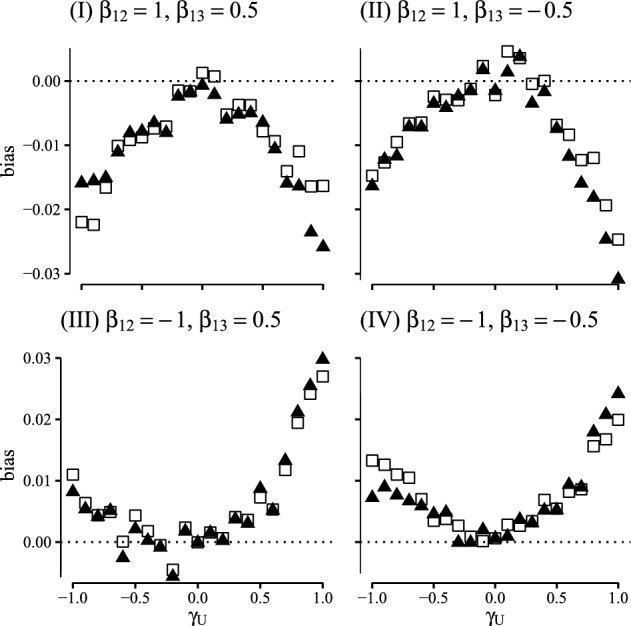
Bias of the plug-in (white rectangles) and one-step (black triangles) estimators of the SDE computed at time points $t=15$ under violation of the identification assumption

We constructed an estimator for the propensity score using a correctly specified logistic regression model. The censoring hazard was estimated using a Cox model with no covariate effects. The remaining nuisance models were estimated using Cox regression models adjusted for main effects of the observed variables. We generated 1000 datasets with a sample size of $n=1000$. For each dataset we computed the plug-in estimator and the one-step estimator for the SDE evaluated at time point $t=15$. The results are depicted in Fig. 5. It is seen that the bias increases with the magnitude of the association with the unmeasured common risk factor *U*. The direction of the bias depends on the effect of treatment on illness: when the treatment has a protective effect on disease the estimator is downwards biased, and when the treatment has a harmful effect on disease the bias is positive.

## Real data application

Using data from the Danish nationwide registries we identified all hospital admissions for first time acute myocardial infarction (MI) between 2010 and 2014. To get a more homogeneous study population we only included patients who were treated with a Percutaneous Coronary Intervention (PCI). We also excluded patients with a preexisting alcohol abuse diagnosis or chronic kidney disease diagnosis and patients younger than 30 years or older than 100 years of age. We set the index date for inclusion at 30 days following discharge and excluded patients who died prior to the index data. We defined the treatment arm as those patients who picked up a prescription for DAPT before the index date and the placebo group as those who did not. Patients who were still alive by the end of 2019 were administratively censored. Among the 16,081 patients in the study population 3856 patients had a recurrent cardiovascular event (defined as a hospital diagnosis of MI, stroke or heart failure) and were subsequently censored, 968 patients died within follow-up without having a recurrent cardiovascular event and 1385 patients experienced a recurrent cardiovascular event and subsequently died within followup.

**Fig. 6:**
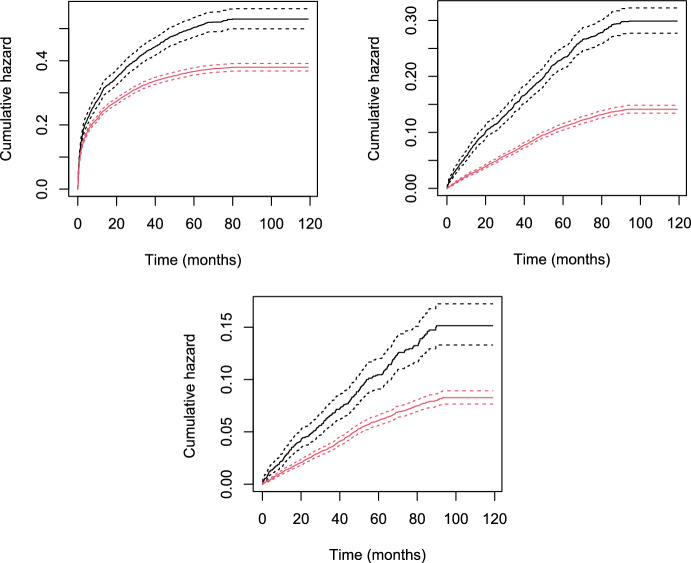
Nelson–Aalen estimates of the cumulative hazards of MI (top left), overall mortality (top right) and death without recurrent MI (bottom) in our cohort. The red curves are the treatment arm and the black curves are the placebo arm. Along with the hazards (solid lines) are shown 95% confidence intervals (dashed lines) (Color figure online)

The cumulative hazard curves in Fig. 6 suggest that treatment reduces both risk of recurrent cardiovascular event, overall mortality and death without recurrent cardiovascular event. To access how much of the effect of DAPT on mortality was mediated through recurrent cardiovascular events we estimated the separable direct and indirect effects. That is, we assume that the treatment has two components that could in principle be manipulated separately: one component $A^I$ which only affects the risk of recurrent cardiovascular event directly and another component $A^D$ which affects mortality through other pathways. A possible interpretation of these treatment components was discussed in Sect. 3. We can then define the separable indirect effect as the effect under an intervention that fixes the treatment component affecting affecting mortality through other pathways than recurrent cardiovascular events but varies the treatment component affecting cardiovascular events. Similarly we can define the separable direct effect as the effect that fixes the treatment component affecting cardiovascular events and varies the component affecting mortality through other pathways.

**Fig. 7:**
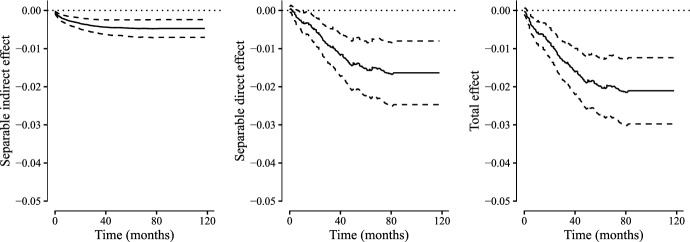
Estimates of the separable direct effect (SDE), separable indirect effect (SIE) and total effect (TE) using the one-step estimator. Solid lines represent effect estimates and dashed lines the corresponding 95 % point-wise confidence intervals

**Fig. 8:**
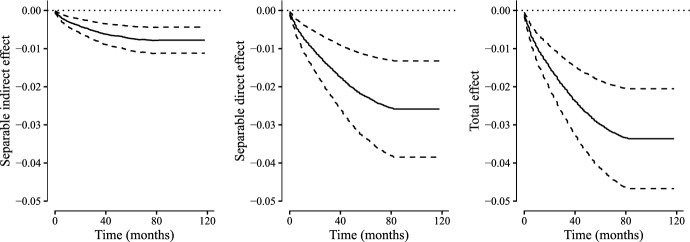
Estimates of the separable direct effect (SDE), separable indirect effect (SIE) and total effect (TE) using the plug-in estimator. Solid lines represent effect estimates and dashed lines the corresponding 95 % point-wise confidence intervals

We estimated the separable effects using the plug-in estimator and the one-step estimator presented in Sect. 4.2. Both estimators used semi-parametric working models for the nuisance parameters. In particular, we used Cox regression models for the three transition hazards. The models were adjusted for baseline age, sex, hypertension diagnosis, prior gastrointestinal bleeding, diabetes, chronic liver disease, cancer, atrial fibrillation, Anemia, prior heart failure or stroke. We computed Wald-type point-wise confidence intervals based on 500 bootstrap data sets.

The results of our analysis are presented in Figs. 7 and 8. In addition to the separable direct and indirect effects we have also depicted the total effect, c.f., Eq. (4).

Our results suggest that the treatment reduces mortality both through recurrent cardiovascular events and through other pathways. That is, within the limitations of our study, we can conclude that the modified treatment that fixes the component affecting mortality through other pathways than recurrent cardiovascular events does not capture the entire protective effect of the treatment. In fact a substantial fraction of the protective effect of DAPT on mortality is a direct effect.

We recognize several potential limitations with our study. First, we likely have confounding by indication in that frail individuals are less likely to be prescribed the treatment. Therefore the drug will appear more effective than it actually is, also on non-cardiovascular mortality. This phenomenon is notoriously difficult to adjust for because of unmeasured confounding. Second, comorbidities such as diabetes status are essentially time-varying covariates. It is a major limitation of our method that we only adjust for baseline covarites. Third, a potential issue is that many cardiovascular events go undetected or are not entered into the registries e.g. when a patient dies suddenly without prior hospital admission. Finally, the overall risk of bleeding, which is the main side effect of DAPT, is very low.

## Discussion

### Relation to other approaches

The main difficulty when formulating causal mediation targets in the illness-death model is that the mediating event is truncated by the terminal event. In this paper we proposed causal mediation estimands using the concept of separable effects, which considers interventions on separate components of the treatment instead of interventions on the mediator. This approach avoids the conceptual issues that arise when the terminal event occurs before the mediator, rendering the mediator undefined. However, this comes at the cost of assuming that the treatment components can be manipulated separately, which may not always be appropriate.

Depending on the causal question at hand there are other approaches in the literature that may be useful for defining mediation targets in the illness-death model.

Valeri et al. (2021) propose randomized interventional direct and indirect effects. Instead of considering manipulations of the mediator, they consider stochastic interventions on the intermediate time-to-event distribution conditional on baseline covariates. The authors then define the ‘stochastic direct effect’ as the difference in survival across exposure groups under a stochastic intervention that fixes the intermediate time-to-event distribution to be the same in both exposure groups. The ‘stochastic indirect effect’ is defined as the difference in survival within an exposure group when the intermediate time-to-event distribution is varied. Their approach result in the same identifying functionals as in our paper, but under different identifiability conditions. Thus the target parameter in our paper can also be interpreted as an interventional effect.

A different alternative is principal stratification which has often been advocated in the presence of truncation (Zhang and Rubin 2003; Comment et al. 2019). A recent paper by Gao et al. (2021) proposes a principal stratification approach for defining causal mediation effects in the subgroup where the intermediate event will happen before the potential terminal event when given either of two treatment options. This strata corresponds to a multistate model where only the transition from the ‘healthy’ state to the ‘illness’ state and from the ‘illness’ state to ‘death’ are involved, an thus their approach leads to a different identifying functional than the one in our paper. This method avoids the issues that arise when death occurs prior to the non-terminal event. However a limitation is that the empirical usefulness of the estimand is debatable since the subgroup for which the estimand is defined can never be observed.

Huang (2021) proposes a method for causal mediation with ‘semicompeting risk data’, based on counterfactual counting processes for the latent intermediate event and the terminal event. To circumvent the undefinability of the intermediate event the author assumes that if the intermediate event does not occur before the terminal event it would never occur within follow-up. The paper was accompanied by a number of commentaries (Stensrud et al. 2021; Fulcher et al. 2021; Chan et al. 2021) which argue that the identification assumptions are too restrictive for most practical contexts. As the authors do not use a classical illness-death model framework, it is not clear to us how their identifying functional is connected to ours.

### Conclusion and possible extensions

In this paper we proposed causal estimands for the separable direct and indirect effects of a baseline exposure on a terminal time-to-event outcome mediated by the illness state of a continuous-time illness-death process. We proposed a plug-in estimator based on the identifying functional, and a one-step estimator which solves the efficient influence function. We showed that the one-step estimator is multiply robust under appropriate regularity conditions, and we confirmed these theoretical properties in a simulation study which showed an impressive performance of the one-step estimator. To illustrate our method we applied the estimators to a Danish registry data set to study how much of the effect of DAPT on mortality was mediated through stroke or MI.

As mentioned in Sect. 6 a major limitation of our work is that we have only considered the case of baseline covariates, ignoring the possibility of changes during the followup period. Such changes are present in most real-word data including the DAPT example. Extending the method to handle time-varying covariates is thus an important topic for future research. We believe that our method can be generalized to allow for covariates measured at random times on a continuous scale similar to the setting considered in Rytgaard et al. (2022). Since the time-varying confounders are potentially affected by treatment one would need to consider a more general notion of separable effects as described in Stensrud et al. (2021) who define separable direct and indirect effects in a discrete-time competing risk model with time-varying common causes of the event of interest and the competing event.

Another important topic for future research is the use of data-adaptive estimation of the nuisance parameters in the illness-death model. Our focus in this paper has been on (semi-)parametric models for the nuisance parameters. However, to avoid misspecification of the nuisance models one may wish to use estimators that are more flexible. The one-step estimator derived in this article is compatible with such data-adaptive estimators, provided certain rate conditions hold.

Finally, in this paper we have limited our attention to the illness-death model where the terminal event death is the outcome of interest. Often the outcome of interest is not a terminal event, and both the mediator and the outcome are subject to competing risk from death. A natural extension of our work is to consider an ‘extended illness-death model’ with a fourth state representing the competing event.

## Appendices

Appendix A: CAR

Appendix B: Proof of Lemma 1 (identifiability)

Appendix C: Derivation of full-data EIF

Appendix D: Derivation of observed-data EIF

Appendix E: Proof of Lemma 2 (multiple robustness of full-data EIF)

Appendix F: Proof of Lemma 3 (multiple robustness of observed-data EIF)

Appendix G: Simulation details

## A CAR

This censoring mechanism induces monotone coarsening (Gill et al. 1997; Tsiatis 2006). Building upon the continuous-time monotone coarsening framework of Tsiatis (2006) Chapter 9.3 we introduce a so-called coarsening variable ${\mathcal {C}}$. The coarsening variable is a continuous random variable which is equal to the censoring time when ${{\tilde{C}}} < T_1$ or $T_1 < {\tilde{C}} \le T_2$, and equal to $\infty$ when the data is uncensored. Let $\tau$ be a time horizon chosen such that there exists $\epsilon >0$ with $P({\tilde{C}}>\tau )>\epsilon >0$. For any time $r \in [0, \tau ]$ we define the set

$$\begin{aligned} \{r \le {\mathcal {C}}< r + \mathop {}\!\textrm{d}r\}= \{r \le {\tilde{C}}< r+ \mathop {}\!\textrm{d}r, {\tilde{C}}< T_1\} \cup \{r \le {\tilde{C}}< r + \mathop {}\!\textrm{d}r, \eta = 1, T_1 < {\tilde{C}} \le T_2\}. \end{aligned}$$

In particular, when ${\mathcal {C}}=r$ we observe the many-to-one mapping

$$\begin{aligned} G_r(Z)= {\left\{ \begin{array}{ll} (T_1 \ge r, T_2\ge r, A, W) &{}\text { if } r< T_1 \\ (\eta =1, T_1, T_1< r, T_2 \ge r, A, W) &{}\text { if } \eta =1 \text { and } T_1 < r \le T_2 \\ (T_1, \eta , T_2, A, W) &{}\text { if } r=\infty \end{array}\right. }, \end{aligned}$$

and the observed data may be expressed as

$$\begin{aligned} O= \left\{ {\mathcal {C}}, G_{{\mathcal {C}}}(Z)\right\} . \end{aligned}$$

The coarsening mechanism is monotone since $G_r(Z) \subseteq G_{r'}(Z)$ for $r>r'$. Following Tsiatis (2006) Chapter 9.3 the CAR assumption is formally defined by

12 $$\begin{aligned} \lambda _{{\mathcal {C}}}(r; G_r(Z)) = \lambda _{{\mathcal {C}}}(r;Z), \end{aligned}$$

where the coarsening hazard may be written

$$\begin{aligned} \lambda _{{\mathcal {C}}}(r;Z) =&\lim _{ \mathop {}\!\textrm{d}r \rightarrow 0} \frac{P(r \le {\mathcal {C}} \le r + \mathop {}\!\textrm{d}r \mid {\mathcal {C}} \ge r, Z)}{\mathop {}\!\textrm{d}r} \\ =&\lim _{\mathop {}\!\textrm{d}r \rightarrow 0} \frac{P({\tilde{C}}\le r+ \mathop {}\!\textrm{d}r, T_1> {\tilde{C}} \mid ({\tilde{C}}\ge r, T_1> {\tilde{C}}) \cup ({\tilde{C}} \ge r, T_1< {\tilde{C}} \le T_2) \cup (T_2< {\tilde{C}}), Z)}{\mathop {}\!\textrm{d}r}\\&+ \lim _{\mathop {}\!\textrm{d}r \rightarrow 0} \frac{P({\tilde{C}}\le r+\mathop {}\!\textrm{d}r, T_1< {\tilde{C}} \le T_2 \mid ({\tilde{C}}\ge r, T_1> {\tilde{C}}) \cup ({\tilde{C}} \ge r, T_1< {\tilde{C}} \le T_2) \cup (T_2< {\tilde{C}}), Z)}{\mathop {}\!\textrm{d}r}\\ =&I(T_1> r) \underbrace{\lim _{\mathop {}\!\textrm{d}r \rightarrow 0} \frac{P({\tilde{C}}\le r+\mathop {}\!\textrm{d}r, T_1 > {\tilde{C}} \mid {\tilde{C}}\ge r, Z)}{\mathop {}\!\textrm{d}r}}_{:=\alpha _{{\tilde{C}},1}(r; Z)} \\&+ I(T_1<r \le T_2) \underbrace{\lim _{\mathop {}\!\textrm{d}r \rightarrow 0} \frac{P({\tilde{C}}\le r+\mathop {}\!\textrm{d}r, T_1< {\tilde{C}} \le T_2 \mid T_1 < r \le {\tilde{C}}, Z)}{\mathop {}\!\textrm{d}r}}_{:=\alpha _{{\tilde{C}},2}(r; Z)} . \end{aligned}$$

That is, if we assume (12),

$$\begin{aligned} I(T_1>r)\alpha _{{\tilde{C}},1}(r; Z)&= I(T_1>r) \alpha _{{\tilde{C}},1}(r \mid A, W ), \\ I(T_1< r \le T_2) \alpha _{{\tilde{C}},2}(r; Z)&=I(T_1 < r \le T_2) \alpha _{{\tilde{C}},2}(r \mid T_1, A, W), \end{aligned}$$

where

$$\begin{aligned} \alpha _{{\tilde{C}},1}(r \mid A, W)&= \lim _{\mathop {}\!\textrm{d}r \rightarrow 0} \frac{P({\tilde{C}}\le r+\mathop {}\!\textrm{d}r, T_1 > {\tilde{C}} \mid {\tilde{T}}_2\ge r, A, W)}{\mathop {}\!\textrm{d}r}, \\ \alpha _{{\tilde{C}},2}(r \mid T_1, A, W)&= \lim _{\mathop {}\!\textrm{d}r \rightarrow 0} \frac{P({\tilde{C}}\le r+\mathop {}\!\textrm{d}r, T_1< {\tilde{C}} \le T_2 \mid T_1 < r \le {\tilde{T}}_2, T_1, A, W)}{\mathop {}\!\textrm{d}r}. \end{aligned}$$

## B Proof of Lemma 1

All transition probabilities of the illness-death model can be expressed in terms of the hazards for the transitions (see e.g. Putter et al. (2007)). For instance, the probability of going from state 1 directly to state 3, within a time interval (*s*, *t*], can be expressed as

$$\begin{aligned} Pr(T_2 \le t, \eta =0 \mid T_1 > s)=\int _{s}^{t} \exp \left[ -\int _s^r \left\{ \lambda _{12}(u) +\lambda _{13}(u)\right\} \mathop {}\!\textrm{d}u \right] \lambda _{13}(r) \mathop {}\!\textrm{d}r, \end{aligned}$$

where we have omitted the baseline covariates for now.

The probability of going from state 1 to state 3 moving through state 2, within a time interval (*s*, *t*], can be expressed as

$$\begin{aligned} Pr(T_2 \le t, \eta =1 \mid T_1>s)&= \int _{s}^{t}\left[ \int _r^t \exp \left\{ -\int _r^s \lambda _{23}(u, u-r) \mathop {}\!\textrm{d}u\right\} \lambda _{23}(s, s-r) \mathop {}\!\textrm{d}s \right] \\&\quad \exp \left[ -\int _s^r \left\{ \lambda _{12}(u) +\lambda _{13}(u)\right\} \mathop {}\!\textrm{d}u \right] \lambda _{12}(r) \mathop {}\!\textrm{d}r. \end{aligned}$$

Then

13 $$\begin{aligned} \begin{aligned} Pr(T_2 \le t) =&Pr(T_2 \le t, \eta =0 \mid T_1> 0) + Pr(T_2 \le t, \eta =1 \mid T_1>0)\\ =&\int _{0}^{t} \exp \left[ -\int _0^r \left\{ \lambda _{12}(u) +\lambda _{13}(u)\right\} \mathop {}\!\textrm{d}u \right] \left\{ \lambda _{12}(r) + \lambda _{13}(r)\right\} \mathop {}\!\textrm{d}r \\&-\int _{0}^{t} \exp \left\{ -\int _r^t \lambda _{23}(u, u-r) \mathop {}\!\textrm{d}u\right\} \exp \left[ -\int _0^r \left\{ \lambda _{12}(u) +\lambda _{13}(u)\right\} \mathop {}\!\textrm{d}u \right] \lambda _{12}(r) \mathop {}\!\textrm{d}r \\ =&1 - \exp \left[ -\int _0^t \left\{ \lambda _{12}(u) +\lambda _{13}(u)\right\} \mathop {}\!\textrm{d}u \right] \\&-\int _{0}^{t} \exp \left\{ -\int _r^t \lambda _{23}(u, u-r) \mathop {}\!\textrm{d}u\right\} \exp \left[ -\int _0^r \left\{ \lambda _{12}(u) +\lambda _{13}(u)\right\} \mathop {}\!\textrm{d}u \right] \lambda _{12}(r) \mathop {}\!\textrm{d}r. \end{aligned} \end{aligned}$$

where the last equality follows using $\int _r^t \exp \left\{ -\int _r^s \lambda _{23}(u, u-r) \mathop {}\!\textrm{d}u\right\} \lambda _{23}(s, s-r) \mathop {}\!\textrm{d}s =1-\exp \left\{ -\int _r^t \lambda _{23}(u, u-r) \mathop {}\!\textrm{d}u\right\}$.

Then

$$\begin{aligned} E\left\{ I(T_2^{a^D,a^{I}}\le \tau )\right\} =&E\left[ E\left\{ I(T_2^{a^D,a^{I}}\le \tau ) \mid W \right\} \right] \\ =&E\Bigg [ 1 -\exp \left\{ -\Lambda _{12}^{a^D,a^{I}}(\tau |W)-\Lambda _{13}^{a^D,a^{I}}(\tau |W)\right\} \\&-\int _{0}^{\tau } \exp \left\{ -\Lambda _{23}^{a^D,a^{I}}(\tau ,\tau -r|W)\right\} \exp \left\{ -\Lambda _{12}^{a^D,a^{I}}(r|W)-\Lambda _{13}^{a^D,a^{I}}(r|W)\right\} \mathop {}\!\textrm{d}\Lambda _{12}^{a^D,a^{I}}(r|W)\Bigg ] \\ =&E\Bigg [1- \exp \left\{ -\Lambda _{12}^{a^I,a^{I}}(\tau |W)-\Lambda _{13}^{a^D,a^D}(\tau |W)\right\} \\&-\int _{0}^{\tau } \exp \left\{ -\Lambda _{23}^{a^D,a^{D}}(\tau ,\tau -r|W)\right\} \exp \left\{ -\Lambda _{12}^{a^I,a^{I}}(r|W)-\Lambda _{13}^{a^D,a^D}(r|W)\right\} \mathop {}\!\textrm{d}\Lambda _{12}^{a^I,a^{I}}(r|W)\Bigg ] \\ =&E \Bigg [1- \exp \left\{ -\Lambda _{12}(\tau |a^I,W)-\Lambda _{13}(\tau |a^D,W)\right\} \\&-\int _{0}^{\tau } S_2(\tau \mid r, a^D, W) \exp \left\{ -\Lambda _{12}(r|a^I,W)-\Lambda _{13}(r|a^D,W)\right\} \mathop {}\!\textrm{d}\Lambda _{12}(r|a^I,W)\Bigg ]. \end{aligned}$$

The first equality is by the law of iterated expectations. The second equality follows by using the representation in Eq. (13) under an intervention that sets $A^D=a^D$ and $A^I=a^I$. The third equality follows by applying the dismissible components conditions. The last equality follows by applying A.0–A.3.

## C Full data EIF

Let $Q_{\varepsilon }$ be a parametric submodel with parameter $\varepsilon \in {\mathbb {R}}$ which passes through *Q* at $\varepsilon =0$. The corresponding tangent space ${\mathcal {T}}^F$ is the closure of the linear span of the scores of the parametric submodels. Due to the factorization of the probability distribution of the full-data density in (1) we can write this as the orthogonal sum

$$\begin{aligned} {\mathcal {T}}^F = {\mathcal {T}}_1^F \oplus {\mathcal {T}}_2^F \oplus {\mathcal {T}}_3^F \oplus {\mathcal {T}}_4^F \end{aligned}$$

where

$$\begin{aligned} {\mathcal {T}}_1^F&= \left\{ \int \alpha (u, A, W) dM_{13}^F(u, A, W) \text { for all functions } \alpha (u, a, w) \right\} \\ {\mathcal {T}}_2^F&= \left\{ \int \alpha (u, A, W) dM_{12}^F(u, A, W) \text { for all functions } \alpha (u, a, w) \right\} \\ {\mathcal {T}}_3^F&= \left\{ \eta \int \alpha (u, T_1, A, W) dM_{23}^F(u, T_1, A, W) \text { for all functions } \alpha (u, r, a, w) \right\} \\ {\mathcal {T}}_4^F&= \left\{ \alpha (A, W) \in {\mathcal {H}}: E\left[ \alpha (A, W)\right] = 0 \right\} \end{aligned}$$

In particular the score on the parametric submodel can be written

$$\begin{aligned} \ell '_Z(z;0)= \partial \log q(z; \varepsilon ) / \partial \varepsilon \mid _{\varepsilon =0}=&\ell '_W(w;0) + \ell '_{A \mid W}(a \mid w; 0) + \ell _{13}'(t_1, \eta \mid a, w;0) \\&+ \ell _{12}'(t_1, \eta \mid a, w;0) + \ell _{23}'(t_2, t_2-t_1, \eta \mid a, w;0) \end{aligned}$$

where $$\ell '_W(w; \varepsilon )= \partial /\partial \varepsilon \log \mu (w; \varepsilon )$$, $$\ell '_{A \mid W}(w \mid a; \varepsilon )= \partial /\partial \varepsilon \log \pi (a \mid w; \varepsilon )$$ and

$$\begin{aligned} \ell '_{12}(T_1, \eta \mid A, W; 0) =&\frac{\partial }{\partial \varepsilon } \left\{ \eta \log \lambda _{12}(T_1 \mid A, W; \varepsilon ) - \int _0^{T_1} \lambda _{12}(u \mid A, W; \varepsilon ) \mathop {}\!\textrm{d}u \right\} \Bigg \vert _{\varepsilon =0} \\ =&\eta \frac{\frac{\partial }{\partial \varepsilon } \left\{ \lambda _{12}(T_1 \mid A, W, \varepsilon )\right\} \mid _{\varepsilon =0}}{\lambda _{12}(T_1 \mid A, W;0)}\\&- \int \frac{\frac{\partial }{\partial \varepsilon } \left\{ \lambda _{12}(T_1 \mid A, W, \varepsilon )\right\} \mid _{\varepsilon =0}}{\lambda _{12}(T_1 \mid A, W;0)}\lambda _{12}(u \mid A, W) I(T_1>u) \mathop {}\!\textrm{d}u \\ =&\int \frac{\frac{\partial }{\partial \varepsilon } \left\{ \lambda _{12}(s \mid A, W, \varepsilon )\right\} \mid _{\varepsilon =0}}{\lambda _{12}(s \mid A, W;0)}\mathop {}\!\textrm{d}M_{12}^F(s, A, W), \end{aligned}$$

and

$$\begin{aligned} \ell '_{13}(T_1, \eta \mid A, W; 0)&= \frac{\partial }{\partial \varepsilon } \left\{ (1-\eta ) \log \lambda _{13}(T_1 \mid A, W; \varepsilon ) \right. \left. - \int _0^{T_1} \lambda _{13}(u \mid A, W; \varepsilon ) \mathop {}\!\textrm{d}u \right\} \Bigg \vert _{\varepsilon =0} \\&= \int \frac{\frac{\partial }{\partial \varepsilon } \left\{ \lambda _{13}(s \mid A, W, \varepsilon )\right\} \mid _{\varepsilon =0}}{\lambda _{13}(s \mid A, W;0)}\mathop {}\!\textrm{d}M_{13}^F(s, A, W), \end{aligned}$$

and

$$\begin{aligned} \ell '_{23}(T_2, T_2-T_1, \eta \mid A, W; 0) =&\frac{\partial }{\partial \varepsilon } \left\{ \eta \log \lambda _{23}(T_2, T_2-T_1 \mid A, W; \varepsilon )\right. \\&\left. - \eta \int _{T_1}^{T_2} \lambda _{23}(u, u-T_1 \mid A, W; \varepsilon ) \mathop {}\!\textrm{d}u \right\} \Bigg \vert _{\varepsilon =0} \\ =&\eta \int \frac{\frac{\partial }{\partial \varepsilon } \left\{ \lambda _{23}(s, s-T_1 \mid A, W, \varepsilon )\right\} \mid _{\varepsilon =0}}{\lambda _{23}(s, s-T_1 \mid A, W;0)}\mathop {}\!\textrm{d}M_{23}^F(s, T_1. A, W). \end{aligned}$$

By Riesz’ representation theorem the efficient influence function can be characterized as any element ${\tilde{\psi }} \in {\mathcal {T}}^F$ which is a pathwise derivative of the target parameter in the sense that

14 $$\begin{aligned} \frac{\partial \psi (Q_{\varepsilon })}{\partial \varepsilon } \Bigg \vert _{\varepsilon =0} = E\left[ {\tilde{\psi }}, \ell '_Z \right] \end{aligned}$$

for any one-dimensional submodel $Q_{\varepsilon }$ with corresponding score $\ell '_Z$.

Note that under the nonparametric model we have that the full-data tangent space is the entire Hilbert space $L_0^2(Q)$ of measurable, mean-zero functions of *Z* equipped with the covariance inner product. Then any pathwise derivate will trivially be contained in ${\mathcal {T}}^F$. Hence we only need to check that the proposed EIF in (8) satisfies (14).

Consider first the left-hand side of (14). We may write

$$\begin{aligned}&\frac{\partial \psi (Q_{\varepsilon })}{\partial \varepsilon } \Bigg \vert _{\varepsilon =0} \\ =&\int _{{\mathscr {W}}} P_{13}(\tau , a^D, a^I, w) \ell '_W(w;0) d\mu (w) \\&+ \int _{{\mathscr {W}}} \Omega _t(a^D, a^I, w) \left\{ \int _0^\tau \frac{\partial }{\partial \varepsilon } \mathop {}\!\textrm{d}\Lambda _{12}(r \mid a^I, w; \varepsilon ) \mid _{\varepsilon =0} \right\} \mathop {}\!\textrm{d}\mu (w)\\&+ \int _{{\mathscr {W}}} \Omega _t(a^D, a^I, w) \left\{ \int _0^\tau \frac{\partial }{\partial \varepsilon }\mathop {}\!\textrm{d}\Lambda _{13}(r \mid a^D, w; \varepsilon ) \mid _{\varepsilon =0}\right\} \mathop {}\!\textrm{d}\mu (w) \\&-\int _{{\mathscr {W}}} \int _0^\tau S_2(\tau \mid r, a^D, w) \Omega _r(a^D, a^I, w) \frac{\partial }{\partial \varepsilon } \mathop {}\!\textrm{d}\Lambda _{12}(r \mid a^I, w; \varepsilon ) \mid _{\varepsilon =0} \mathop {}\!\textrm{d}\mu (w)\\&+\int _{{\mathscr {W}}} \int _0^\tau S_2(\tau \mid r, a^D, w) \Omega _r(a^D, a^I, w) \left\{ \int _0^r \frac{\partial }{\partial \varepsilon } \mathop {}\!\textrm{d}\Lambda _{12}(s \mid a^I, w; \varepsilon ) \mid _{\varepsilon =0}\right\} \mathop {}\!\textrm{d}\Lambda _{12}(r \mid a^I, w) \mathop {}\!\textrm{d}\mu (w)\\&+\int _{{\mathscr {W}}} \int _0^\tau S_2(\tau \mid r, a^D, w) \Omega _r(a^D, a^I, w) \left\{ \int _0^r \frac{\partial }{\partial \varepsilon }\mathop {}\!\textrm{d}\Lambda _{13}(s \mid a^D, w; \varepsilon )\mid _{\varepsilon =0}\right\} \mathop {}\!\textrm{d}\Lambda _{12}(r \mid a^I, w) \mathop {}\!\textrm{d}\mu (w)\\&-\int _{{\mathscr {W}}} \int _0^\tau S_2(\tau \mid r, a^D, w) \left\{ \int _r^\tau \frac{\partial }{\partial \varepsilon } \mathop {}\!\textrm{d}\Lambda _{23}(s, s-r\mid a^D, w; \varepsilon )\mid _{\varepsilon =0}\right\} \Omega _r(a^D, a^I, w) \mathop {}\!\textrm{d}\Lambda _{12}(r \mid a^I, w) \mathop {}\!\textrm{d}\mu (w) \\ =&\int _{{\mathscr {W}}} P_{13}(\tau , a^D, a^I, w) \ell '_W(w;0) d\mu (w) \\&+ \int _{{\mathscr {W}}}\int _0^\tau h_{12,\tau }(r, a^D, a^I, w) \frac{\partial }{\partial \varepsilon } \mathop {}\!\textrm{d}\Lambda _{12}(r \mid a^I, w; \varepsilon ) \mid _{\varepsilon =0}\mathop {}\!\textrm{d}\mu (w) \\&+\int _{{\mathscr {W}}}\int _0^\tau h_{13,\tau }(r, a^D, a^I, w) \frac{\partial }{\partial \varepsilon } \mathop {}\!\textrm{d}\Lambda _{13}(r \mid a^D, w; \varepsilon ) \mid _{\varepsilon =0}\mathop {}\!\textrm{d}\mu (w) \\&+ \int _{{\mathscr {W}}} \int _0^\tau h_{23,\tau }(r, a^D, a^I, w) \int _r^\tau \mathop {}\!\textrm{d}\Lambda _{12}(r \mid a^D, w) \frac{\partial }{\partial \varepsilon } \mathop {}\!\textrm{d}\Lambda _{23}(u, u-r \mid a^D, w; \varepsilon ) \mid _{\varepsilon =0}\mathop {}\!\textrm{d}\mu (w) \end{aligned}$$

where the second equality follows by changing the order of integration.

Consider now the right-hand side of (14). We have by iterated expectations, and the properties of score functions that

15 $$\begin{aligned}&E\left\{ {\tilde{\psi }}(Z; \tau , a^D, a^I) \ell _Z'(Z;0)\right\} =E\left\{ P_{13}(\tau , a^D, a^I, W) \ell '_W(w;0)\right\} \end{aligned}$$

16 $$\begin{aligned}&+E \Bigg [\frac{I(A=a^I)}{P(A=a^I\mid W)} \int _0^\tau h_{12,\tau }(s, a^D, a^I, W)\frac{dM_{12}^F(s, a^I, W)}{S_1(s \mid a^I, W)}\nonumber \\&\times \left\{ \ell _{12}'(T_1 \mid A, W;0)+\ell _{13}'(T_1, \eta \mid A, W;0)\right\} \Bigg ] \end{aligned}$$

17 $$\begin{aligned}&+E \Bigg [\frac{I(A=a^D)}{P(A=a^D\mid W)} \int _0^\tau h_{13,\tau }(s, a^D, a^I, W)\frac{dM_{13}^F(s, a^D, W)}{S_1(s \mid a^D, W)}\nonumber \\&\times \left\{ \ell _{12}'(T_1 \mid A, W;0)+\ell _{13}'(T_1, \eta \mid A, W;0)\right\} \Bigg ]\ \end{aligned}$$

18 $$\begin{aligned}&+E \Bigg \{\frac{I(A=a^D)}{P(A=a^D\mid W)} \frac{\eta h_{23,\tau }(T_1, a^D, a^I, W)}{S_1(T_1 \mid a^D, W)} \int _{T_1}^\tau \frac{dM_{23}^F(s, T_1,a^I, W)}{S_2(s \mid a^D, W)}\times \ell _{23}'(T_2, \eta \mid T_1, A, W;0)\Bigg \} \end{aligned}$$

Du to the representation of the scores $\ell _{12}$ and $\ell _{13}$ in terms of the full-data martingales, the expectations in (16)–(18) are the covariances of martingale stochastic integrals. They be computed by finding the expectation of the corresponding predictable covariation processes (Fleming and Harrington 1991). In particular, the predictable covariation process of $M_{ij}^F$ with itself is the compensator part of the martingale. The predictable covariation process of $M_{12}^F$ and $M_{13}^F$ is zero because the counting processes $N_{12}$ and $N_{13}$ by definition do not jump simultaneously.

Then we may write (16) as

$$\begin{aligned}&E \Bigg [ \frac{I(A=a^I)}{\pi (a^I \mid W)} \int _0^\tau \frac{h_{12,\tau }(s, a^D, a^I, W)}{S_1(s \mid a^I, W)} \frac{\frac{\partial }{\partial \varepsilon } \left\{ \lambda _{12}(s \mid a^I, W, \varepsilon )\right\} \mid _{\varepsilon =0}}{\lambda _{12}(s \mid a^I, W;0)} I(T_1>s) \lambda _{12}(s \mid a^I, W) \mathop {}\!\textrm{d}s \Bigg ]\\&=E \Bigg [ \frac{E \left\{ I(A=a^I) \mid W \right\} }{\pi (a^I \mid W)} \int _0^\tau \frac{h_{12,\tau }(s, a^D, a^I, W)}{S_1(s \mid a^I, W)} \frac{\partial }{\partial \varepsilon } \left\{ \lambda _{12}(s \mid a^I, W, \varepsilon )\right\} \mid _{\varepsilon =0} E \left\{ I(T_1>s) \mid a^I, W \right\} \mathop {}\!\textrm{d}s \Bigg ]\\&=E \Bigg [\int _0^\tau h_{12,\tau }(s, a^D, a^I, W) \frac{d}{d\varepsilon }\left\{ \mathop {}\!\textrm{d}\Lambda _{12}(s \mid a^I, W; \varepsilon )\right\} \mid _{\varepsilon =0} \Bigg ], \end{aligned}$$

and similarly for (17)

$$\begin{aligned}&E \Bigg [ \frac{I(A=a^D)}{\pi (a^D\mid W)} \int _0^\tau \frac{h_{13,\tau }(s, a^D, a^I, W)}{S_1(s \mid a^D, W)} \frac{\frac{\partial }{\partial \varepsilon } \left\{ \lambda _{13}(T_1 \mid A, W, \varepsilon )\right\} \mid _{\varepsilon =0}}{\lambda _{13}(T_1 \mid A, W;0)}I(T_1>s) \lambda _{13}(s \mid a^D, W) \mathop {}\!\textrm{d}s\Bigg ]\\&\quad =E \Bigg [\int _0^\tau h_{13,\tau }(s, a^D, a^I, W) \frac{d}{d\varepsilon }\left\{ \mathop {}\!\textrm{d}\Lambda _{13}(s \mid a^D, W; \varepsilon )\right\} \mid _{\varepsilon =0} \Bigg ]. \end{aligned}$$

Finally we may rewrite (18) as

$$\begin{aligned}&E \Bigg [ \frac{I(A=a^D)}{\pi (a^D \mid W)} \frac{\eta h_{23,\tau }(T_1, a^D, a^I, W)}{S_1(T_1 \mid a^D, W)} \\&\quad \int _{T_1}^\tau \frac{I(T_1<s<T_2)}{S_2(s \mid T_1, a^D, W)} \frac{\frac{\partial }{\partial \varepsilon } \left\{ \lambda _{23}(s, s-T_1 \mid a^D, W, \varepsilon )\right\} \mid _{\varepsilon =0}}{\lambda _{23}(s, s-T_1 \mid a^D, W;0)}\lambda _{23}(s, s-T_1 \mid a^D, W) \mathop {}\!\textrm{d}s \Bigg ]\\&\quad =E \Bigg [ \frac{E \left\{ I(A=a^D) \mid W\right\} }{\pi (a^D \mid W)} E \Bigg \{\frac{\eta h_{23,\tau }(T_1, a^D, a^I, W)}{S_1(T_1 \mid a^D, W)}\\&\quad \int _{T_1}^\tau \frac{E\left\{ I(T_1<s<T_2) \mid T_1, \eta , a^D, W\right\} }{S_2(s \mid T_1, a^D, W)} \\&\quad \times \frac{\partial }{\partial \varepsilon } \left\{ \lambda _{23}(s, s-T_1 \mid a^D, W, \varepsilon )\right\} \mid _{\varepsilon =0}\mathop {}\!\textrm{d}s \mid a^D, W \Bigg \} \Bigg ]\\&\quad =\int _0^\tau h_{23,\tau }(r, a^D, a^I, W) \int _r^\tau \frac{d}{d\varepsilon }\left\{ \mathop {}\!\textrm{d}\Lambda _{23}(u, u-r\mid a^D, w; \varepsilon )\right\} \mid _{\varepsilon =0} \mathop {}\!\textrm{d}\Lambda _{12}(r \mid a^D, W). \end{aligned}$$

Hence we have shown that the proposed influence function is in fact the efficient full-data influence function.

## D Observed-data EIF

By Tsiatis (2006) theorem 10.1 and 10.4 we can map the full-data EIF to the observed-data EIF using the linear operator ${\mathscr {J}}:L_0^2(Q) \rightarrow L_0^2(P)$ which is defined by

19 $$\begin{aligned} {\mathscr {J}}({\tilde{\psi }}(Z; \tau , a^D, a^I))=\frac{\delta {\tilde{\psi }}(Z; \tau , a^D, a^I)}{{K_{\tilde{C}}\{{\tilde{T}}_2,G_{{\tilde{T}}_2}(Z)\}}}+\int \frac{E\{{\tilde{\psi }}(Z; , a^D, a^I)\mid G_u(Z)\}}{{K_{{{\tilde{C}}}}\{u,G_u(Z)\}}} \mathop {}\!\textrm{d}M_{{{\tilde{C}}}}\{u,G_u(Z)\}. \end{aligned}$$

where

$$\begin{aligned} K_{{{\tilde{C}}}}\{u,G_u(Z)\}:=\exp \left[ -\int _0^u \lambda _{\tilde{C}}\left\{ u; G_u(Z)\right\} \mathop {}\!\textrm{d}u\right] . \end{aligned}$$

Using the following lemma we can rewrite the efficient influence function in terms of the observed-data martingales

### Lemma A.1

for any element $\int h(u, Z) \mathop {}\!\textrm{d}M^F(u, Z)\in {\mathscr {T}}^F_i$, for $i=1,2,3$, it holds that

$$\begin{aligned}&{\mathscr {J}}\left\{ \int h(u, Z) \mathop {}\!\textrm{d}M^F(u, Z)\right\} = \int h(u, Z) \frac{\mathop {}\!\textrm{d}M(u, Z)}{K_{{\tilde{C}}}(u, Z)}. \end{aligned}$$

### Proof

We prove the lemma for $i=1$. The remaining cases follow by similar calculations.

First note that given $(T_1 \wedge T_2 \ge u, A, W)$, $M_{12}^F(v, A, W)$ is zero-mean martingale for $v \ge u$, and hence $E\left[ \mathop {}\!\textrm{d}M_{12}^F(v, A, W) \; \Bigg |\;T_1 \wedge T_2 \ge u, A, W \right] = 0$ for $v \ge u$. For $v < u$ we have that, given $(T_1 \wedge T_2 \ge u, A, W)$, $N_{12}(v) = 0$ and $E[I(T_1> v, T_2 >v) \mid T_1 \wedge T_2 \ge u, A, W] = 1$, and hence we can write

20 $$\begin{aligned} E\left[ \mathop {}\!\textrm{d}M_{12}^F(v, X) \; \Bigg |\;T_1 \wedge T_2 \ge u, A, W\right] = -I(v < u )\lambda _{12}(v \mid A, W) \mathop {}\!\textrm{d}v. \end{aligned}$$

Also, for the second term we note that $M^F_{12}$ is fixed given $T_1$ and *A*, *W*, so

21 $$\begin{aligned} E\left[ \mathop {}\!\textrm{d}M_{12}^F(v, X) \; \Bigg |\;\eta = 1, T_1< u < T_2, T_1, A, W \right] = \mathop {}\!\textrm{d}M_{12}(v, A, W). \end{aligned}$$

Then, using (20) and (21), we can write

22 $$\begin{aligned} \begin{aligned}&\int \frac{E\left[ \int h(v, Z) \mathop {}\!\textrm{d}M_{12}^F(v, Z) \; \Bigg |\;G_u(Z) \right] \mathop {}\!\textrm{d}M_{{\tilde{C}}}(u, Z) }{K_{{\tilde{C}}}(u, Z)} \\ =&\int _0^{{{\tilde{T}}}_1} \frac{E\left[ \int h(v, Z) \mathop {}\!\textrm{d}M_{12}^F(v, Z) \; \Bigg |\;G_u(Z) \right] \mathop {}\!\textrm{d}M_{{\tilde{C}}}(u, Z) }{K_{{\tilde{C}}}(u, G_u(Z))}\\&+ \eta \int _{T_1}^{{{\tilde{T}}}_2} \frac{E\left[ \int h(v, Z) \mathop {}\!\textrm{d}M_{12}^F(v, Z) \; \Bigg |\;G_u(Z) \right] \mathop {}\!\textrm{d}M_{{\tilde{C}}}(u, Z) }{K_{{\tilde{C}}}(u, G_u(Z))} \\ =&- \int _0^{{{\tilde{T}}}_1} \int _0^v h(v, X) \mathop {}\!\textrm{d}\Lambda _{12}(v, X) \frac{\mathop {}\!\textrm{d}M_{{\tilde{C}}}(u, Z) }{K_{{\tilde{C}}}(u, G_u(Z))} \\&+ \tilde{\eta }\int h(u, X) \mathop {}\!\textrm{d}M_{12}^F(u, Z) \int _{T_1}^{{{\tilde{T}}}_2} \frac{\mathop {}\!\textrm{d}M_{{\tilde{C}}}(u, Z) }{K_{{\tilde{C}}}(u, G_u(Z))}. \end{aligned} \end{aligned}$$

With similar calculations as in Lu and Tsiatis (2008) Lemma A.2 the first term equals

$$\begin{aligned}&- \frac{(1-{{\tilde{\eta }}})(1-\delta )}{K_{{\tilde{C}}}({{\tilde{T}}}_1 G_{{{\tilde{T}}}_1}(Z))} \int _0^{{{\tilde{T}}}_1} h(u, X) \mathop {}\!\textrm{d}\Lambda _{12}(u, X) + \int _0^{{{\tilde{T}}}_1} \int _u^{{{\tilde{T}}}_1}\frac{\mathop {}\!\textrm{d}\Lambda _{{{\tilde{C}}}}(s; G_s(Z))}{K_{{\tilde{C}}}(s, G_{s}(Z))} h(u, X) \mathop {}\!\textrm{d}\Lambda _{12}(u, X) \\&= - \frac{(1-{{\tilde{\eta }}})(1-\delta )}{K_{{\tilde{C}}}({{\tilde{T}}}_1 G_{{{\tilde{T}}}_1}(Z))} \int _0^{{{\tilde{T}}}_1} h(u, X) \mathop {}\!\textrm{d}\Lambda _{12}(u, X) \\&\quad + \frac{1}{K_{{\tilde{C}}}({{\tilde{T}}}_1, G_{{{\tilde{T}}}_1}(Z))} \int _0^{{{\tilde{T}}}_1} h(u, X) \mathop {}\!\textrm{d}\Lambda _{12}(u, X) - \int _0^{{{\tilde{T}}}_1} \frac{h(u, X)}{K_{{\tilde{C}}}(u, G_u(Z))} \mathop {}\!\textrm{d}\Lambda _{12}(u, X) \\&= \frac{{{\tilde{\eta }}}}{K_{{\tilde{C}}}({{{\tilde{T}}}_1}, G_{\tilde{T}_1}(Z))} \int _0^{{{\tilde{T}}}_1} h(u, X) \mathop {}\!\textrm{d}\Lambda _{12}(u, X) - \int _0^{{{\tilde{T}}}_1} \frac{h(u, X)}{K_{{\tilde{C}}}(u, G_u(Z))} \mathop {}\!\textrm{d}\Lambda _{12}(u, X) \\&\quad + \frac{\delta (1-{{\tilde{\eta }}})}{K_{{\tilde{C}}}({{\tilde{T}}}_1, G_{{{\tilde{T}}}_1}(Z))} \int _0^{{{\tilde{T}}}_1} h(u, X) \mathop {}\!\textrm{d}\Lambda _{12}(u, X) \\&= - \frac{{{\tilde{\eta }}}}{K_{{\tilde{C}}}({{\tilde{T}}}_1, G_{\tilde{T}_1}(Z))} \int _0^{{{\tilde{T}}}_1} h(u, X) \mathop {}\!\textrm{d}M_{12}^F(u, X) + \int _0^{{{\tilde{T}}}_1} \frac{h(u, X)}{K_{{\tilde{C}}}(u, G_u(Z))} \mathop {}\!\textrm{d}M_{12}(u, X) \\&\quad + \frac{\delta (1-{{\tilde{\eta }}})}{K_{{\tilde{C}}}({{\tilde{T}}}_1, G_{{{\tilde{T}}}_1}(Z))} \int _0^{{{\tilde{T}}}_1} h(u, X) \mathop {}\!\textrm{d}\Lambda _{12}(u, X) \end{aligned}$$

The second term in (22) equals

$$\begin{aligned}&{{\tilde{\eta }}} \int h(u, X) \mathop {}\!\textrm{d}M_{12}^F(u, Z) \int _{T_1}^{{{\tilde{T}}}_2} \frac{\mathop {}\!\textrm{d}M_{{{\tilde{C}}}}(u, Z) }{K_{{\tilde{C}}}(u, G_u(Z))} \\&= {{\tilde{\eta }}} \int h(u, X) \mathop {}\!\textrm{d}M_{12}^F(u, Z) \left[ \frac{1-\delta }{K_{{\tilde{C}}}({{\tilde{T}}}_2, G_{{{\tilde{T}}}_2}(Z))} - \left( \frac{1}{K_{{\tilde{C}}}({{\tilde{T}}}_2, G_{{{\tilde{T}}}_2}(Z))} - \frac{1}{K_{{\tilde{C}}}(T_1, G_{T_1}(Z))} \right) \right] \\&= {{\tilde{\eta }}} \int h(u, X) \mathop {}\!\textrm{d}M_{12}^F(u, Z) \left[ \frac{1}{K_{{\tilde{C}}}(T_1, G_{T_1}(Z))} -\frac{\delta }{K_{{\tilde{C}}}({\tilde{T}}_2, G_{{{\tilde{T}}}_2}(Z))} \right] . \end{aligned}$$

Using that ${{\tilde{T}}}_1 = {{\tilde{T}}}_2$ and $\Lambda _{12} = - M^F_{12}$ when $\delta (1-{{\tilde{\eta }}})=1$ and $T_1 = {{\tilde{T}}}_1$ when $\eta =1$, adding the two final lines of the two previous displays gives

$$\begin{aligned} \int _0^{{{\tilde{T}}}_1} \frac{h(u, X)}{K_{{\tilde{C}}}(u, G_u(Z))} \mathop {}\!\textrm{d}M_{12}(u, X) - \frac{\delta }{K_{{\tilde{C}}}({{\tilde{T}}}_2, G_{\tilde{T}_2}(Z))} \int _0^{{{\tilde{T}}}_2} h(u, X) \mathop {}\!\textrm{d}M^F_{12}(u, X), \end{aligned}$$

which is the desired result. $\square$

## E Proof of Lemma 2

By iterated expectations, it follows that

23 $$\begin{aligned} \begin{aligned}&E\left[ \frac{I(A=a^{I})}{\pi ^*(a^{I}\mid W)} \int _0^\tau \frac{h^*_{12}(s, a^D, a^I, W)dM^{F*}_{12}(s, a^I, W)}{S^*_1(s \mid a^I, W)}\right] \\&\quad =E\left[ \frac{\pi (a^{I} \mid W)}{\pi ^*(a^{I}\mid W)} \right. \\&\quad \left. \int _0^\tau \frac{h^*_{12}(s, a^D, a^I, W)S_1(s \mid a^I, W)}{S^*_1(s \mid a^I, W)} \left\{ \mathop {}\!\textrm{d}\Lambda _{12}(s \mid a^I, W)-\mathop {}\!\textrm{d}\Lambda _{12}^*(s \mid a^I, W) \right\} \right] , \end{aligned} \end{aligned}$$

24 $$\begin{aligned} \begin{aligned}&E\left[ \frac{I(A=a^D)}{\pi ^*(a^D \mid W)} \int _0^\tau \frac{ h^*_{13}(s, a^D, a^I, W) dM^{F*}_{13}(s, a^D, W)}{S^*_1(s \mid a^D, W)} \right] \\&\quad =E\left[ \frac{\pi (a^{D} \mid W)}{\pi ^*(a^{D}\mid W)}\right. \\&\quad \left. \int _0^\tau \frac{h_{13,}^*(s, a^D, a^I, W)S_1(s \mid a^D, W)}{S^*_1(s \mid a^D, W)} \left\{ \mathop {}\!\textrm{d}\Lambda _{13}(s \mid a^D, W)-\mathop {}\!\textrm{d}\Lambda _{13}^*(s \mid a^D, W) \right\} \right] , \end{aligned} \end{aligned}$$

and

25 $$\begin{aligned} \begin{aligned}&E\left[ \frac{I(A=a^D)}{\pi ^*(a^D \mid W)} \frac{\eta h^*_{23}(T_1, a^D, a^I, W)}{S^*_1(T_1 \mid a^D, W)} \int _{T_1}^\tau \frac{ dM^{F*}_{23}(s, a^D, T_1, W)}{S^*_2(s \mid T_1, a^D, W)} \right] \\&\quad =E\left[ \frac{\pi (a^D \mid W)}{\pi ^*(a^{D}\mid W)} E \left\{ \frac{h^*_{23}(T_1, a^D, a^I, W)}{S^*_1(T_1 \mid a^D, W)} \int _{T_1}^\tau \frac{S_2(s \mid T_1, a^D, W)}{S^*_2(s \mid T_1, a^D, W)} \left\{ \mathop {}\!\textrm{d}\Lambda _{23}(s \mid T_1,a^D, W)\right. \right. \right. \\&\quad \left. \left. \left. -\mathop {}\!\textrm{d}\Lambda _{23}^*(s \mid T_1, a^D, W) \right\} \Big \vert a^D, W \right\} \right] \\&\quad =E\left[ \frac{\pi (a^D \mid W)}{\pi ^*(a^{D}\mid W)} \int _0^\tau \frac{h^*_{23}(s, a^D, a^I, W)}{S^*_1(s \mid a^D, W)} \left\{ 1-\frac{S_2(\tau \mid s, a^D, W)}{S_2^*(\tau \mid s, a^D, W)} \right\} \right. \\&\quad \left. S_1(s \mid a^D, W) \mathop {}\!\textrm{d}\Lambda _{12}(s \mid a^D, W) \right] \end{aligned} \end{aligned}$$

Suppose $\pi$, $\Lambda _{13}$ and $\Lambda _{23}$ are correctly specified, but $\Lambda _{12}$ is not. Then the terms (24) and (25) are zero, and we have

$$\begin{aligned}&E \left[ {\tilde{\psi }}(Q^*)(Z, a^D, a^I, W)\right] \\&=E\Bigg [ e^{-\Lambda _{12}^*( \tau \mid a^I, W)- \Lambda _{13}(\tau \mid a^D, W)}\int _0^\tau e^{-\Lambda _{12}(s \mid a^I, W)+ \Lambda _{12}^*(s \mid a^I, W)} \left\{ \mathop {}\!\textrm{d}\Lambda _{12}(s \mid a^I, W)\right. \\&\quad \left. -\mathop {}\!\textrm{d}\Lambda _{12}^*(s \mid a^I, W) \right\} \Bigg ]\\&-E\Bigg [\int _0^\tau S_2(\tau \mid s, a^D, W)\Omega _s(a^D, a^I, W) \left\{ \mathop {}\!\textrm{d}\Lambda _{12}(s \mid a^I, W)-\mathop {}\!\textrm{d}\Lambda _{12}^*(s \mid a^I, W) \right\} \Bigg ] \\&+E\Bigg [\int _0^\tau \left\{ \int _0^s e^{-\Lambda _{12}(s \mid a^I, W)+ \Lambda _{12}^*(s \mid a^I, W)}\left\{ \mathop {}\!\textrm{d}\Lambda _{12}(s \mid a^I, W)-\mathop {}\!\textrm{d}\Lambda _{12}^*(s \mid a^I, W) \right\} \right\} \\&\quad S_2(\tau \mid s a^D, W)\\&\quad \times e^{-\Lambda _{12}^*(s \mid a^I, W)- \Lambda _{13}(s \mid a^D, W)} \mathop {}\!\textrm{d}\Lambda _{12}^*(s \mid a^I, W) \Bigg ] \\&\quad + E\Bigg [1- e^{-\Lambda _{12}^*(\tau \mid a^I, W)- \Lambda _{13}(\tau \mid a^D, W)} \\&\quad - \int _0^\tau S_2(\tau \mid s, a^D, W)e^{-\Lambda _{12}^*(s \mid a^I, W)- \Lambda _{13}(s \mid a^D, W)} \mathop {}\!\textrm{d}\Lambda _{12}^*(s \mid a^I, W) \Bigg ]\\&\quad - \psi (Q^*; \tau , a^D, a^I) \\&\quad =E\Bigg [ e^{-\Lambda _{12}^*(\tau \mid a^I, W)- \Lambda _{13}(\tau \mid a^D, W)}\left\{ 1-e^{-\Lambda _{12}(\tau \mid a^I, W)+ \Lambda _{12}^*(\tau \mid a^I, W)} \right\} \Bigg ]\\&\quad -E\Bigg [\int _0^\tau S_2(\tau \mid s, a^D, W)\Omega _s(a^D, a^I, W) \left\{ \mathop {}\!\textrm{d}\Lambda _{12}(s \mid a^I, W)-\mathop {}\!\textrm{d}\Lambda _{12}^*(s \mid a^I, W) \right\} \Bigg ] \\&\quad +E\Bigg [\int _0^\tau \left\{ 1-e^{-\Lambda _{12}(s \mid a^I, W)+ \Lambda _{12}^*(s \mid a^I, W)} \right\} \\&\quad S_2(\tau \mid s, a^D, W) e^{-\Lambda _{12}^*(s \mid a^I, W)- \Lambda _{13}(s \mid a^D, W)} \mathop {}\!\textrm{d}\Lambda _{12}^*(s \mid a^I, W) \Bigg ] \\&\quad + E\Bigg [1- e^{-\Lambda _{12}^*(\tau \mid a^I, W)- \Lambda _{13}(\tau \mid a^D, W)}\\&\quad - \int _0^\tau S_2(\tau \mid s, a^D, W)e^{-\Lambda _{12}^*(s \mid a^I, W)- \Lambda _{13}(s \mid a^D, W)} \mathop {}\!\textrm{d}\Lambda _{12}^*(s \mid a^I, W) \Bigg ]\\&\quad - \psi (Q^*; \tau , a^D, a^I) \\&\quad = E\Bigg [1- \Omega _t(a^D, a^I, W) + \int _0^\tau S_2(\tau \mid s, a^D, W)\Omega _s(a^D, a^I, W)\mathop {}\!\textrm{d}\Lambda _{12}(s \mid a^I, W)\Bigg ]\\&\quad - \psi (Q^*; \tau , a^D, a^I) \\&\quad = \psi (Q; \tau , a^D, a^I)- \psi (Q^*; \tau , a^D, a^I) \end{aligned}$$

Similarly, suppose $\pi$, $\Lambda _{12}$ and $\Lambda _{23}$ are correctly specified, but $\Lambda _{13}$ is not. Then (23) and (25) are 0, and

$$\begin{aligned}&E \left[ {\tilde{\psi }}(Q^*)(Z, a^D, a^I, W)\right] \\&\quad =E\Bigg [ e^{-\Lambda _{12}(\tau \mid a^I, W)- \Lambda _{13}^*(\tau \mid a^D, W)}\left\{ 1-e^{-\Lambda _{13}(\tau \mid a^I, W)+ \Lambda _{13}^*(\tau \mid a^I, W)} \right\} \Bigg ]\\&\quad +E\Bigg [\int _0^\tau \left\{ 1-e^{-\Lambda _{13}(s \mid a^I, W)+ \Lambda _{13}^*(s \mid a^I, W)} \right\} \\&\quad S_2(\tau \mid s, a^D, W) e^{-\Lambda _{12}(s \mid a^I, W)- \Lambda _{13}^*(s \mid a^D, W)} \mathop {}\!\textrm{d}\Lambda _{12}(s \mid a^I, W) \Bigg ] \\&\quad + E\Bigg [1- e^{-\Lambda _{12}(\tau \mid a^I, W)- \Lambda _{13}^*(\tau \mid a^D, W)} \\&\quad - \int _0^\tau S_2(\tau \mid s, a^D, W)e^{-\Lambda _{12}(s \mid a^I, W)- \Lambda _{13}^*(s \mid a^D, W)} \mathop {}\!\textrm{d}\Lambda _{12}(s \mid a^I, W) \Bigg ]\\&\quad - \psi (Q^*; \tau , a^D, a^I) \\&\quad = E\Bigg [1- \Omega _t(a^D, a^I, W) + \int _0^\tau S_2(\tau \mid s, a^D, W)\Omega _s(a^D, a^I, W)\mathop {}\!\textrm{d}\Lambda _{12}(s \mid a^I, W)\Bigg ]- \psi (Q^*; \tau , a^D, a^I) \\&\quad =\psi (Q; \tau , a^D, a^I)-\psi (Q^*; \tau , a^D, a^I) \end{aligned}$$

Finally, suppose $\pi$, $\Lambda _{12}$ and $\Lambda _{13}$ are correctly specified, but $\Lambda _{23}$ is not. Then (23) and (24) are 0, and

$$\begin{aligned}&E \left[ {\tilde{\psi }}(Q^*)(Z, a^D, a^I, W)\right] \\&\quad =E \left[ \int _0^\tau S_2^*(\tau \mid s, a^D, W)\left\{ 1- \frac{S_2(\tau \mid s, a^D, W)}{S_2^*(\tau \mid s, a^D, W)} \right\} \Omega _s(a^D, a^I, W) \mathop {}\!\textrm{d}\Lambda _{12}(s \mid a^I, W) \right] \\&\quad + E\Bigg [1- \Omega _t(a^D, a^I, W) - \int _0^\tau S_2^*(\tau \mid s, a^D, W)\Omega _s(a^D, a^I, W) \mathop {}\!\textrm{d}\Lambda _{12}(s \mid a^I, W) \Bigg ]\\&\quad - \psi (Q^*; \tau , a^D, a^I) \\&\quad =\psi (Q; \tau , a^D, a^I)-\psi (Q^*; \tau , a^D, a^I) \end{aligned}$$

## F Proof of Lemma 3

Using the representation of the full-data influence function in (19) we need to show

26 $$\begin{aligned}&E \left[ \int \frac{\left\{ {\tilde{\psi }}(Z; \tau , a^D, a^I) - E^*\{{\tilde{\psi }}(Z; \tau , a^D, a^I) \mid G_s(Z)\}\right\} }{K_{{{\tilde{C}}}}^*(s\mid G_s(Z))} \mathop {}\!\textrm{d}M^*_ {\tilde{C}}(s, G_s(Z)) \right] =0 \end{aligned}$$

when either $\Lambda _{{{\tilde{C}}}}$ is correctly specified or the entire outcome distribution, that is $\Lambda _{12}$, $\Lambda _{13}$ and $\Lambda _{23}$, are correctly specified.

By iterated expectations (26) holds if we show

- $E\left[ \mathop {}\!\textrm{d}M^*_ {{{\tilde{C}}}}(s, G_s(Z))\mid Z \right] =0$ when $\Lambda _{{{\tilde{C}}}}$ is correctly specified
- $E\{{\tilde{\psi }}(Z; \tau , a^D, a^I) \mid G_u(Z)\} - E^*\{{\tilde{\psi }}(Z; \tau , a^D, a^I) \mid G_u(Z)\}=0$ if $\Lambda _{12}$, $\Lambda _{13}$ and $\Lambda _{23}$ are correctly specified

(a)

Note that, under CAR, we have

$$\begin{aligned}&E\left\{ \mathop {}\!\textrm{d}N_{{\tilde{C}}}(u)\mid T_1, T_2, \eta , A, W \right\} \\&\quad = E\left\{ I({{\tilde{C}}}= u, T_1 \ge u) \mid T_1 , T_2, \eta , A, W \right\} + E\left\{ \eta I({{\tilde{C}}}= u, T_1 \le u< T_2) \mid T_1, T_2, \eta , A, W \right\} \\&\quad =I(u \le T_1)K_{{{\tilde{C}}},1}( u \mid A, W)\alpha _{{{\tilde{C}}},1}(u \mid A, W) \\&\quad + I(T_1 \le u < T_2)K_{{{\tilde{C}}},2}(u \mid T_1, A, W)\alpha _{{{\tilde{C}}},2}(u \mid T_1, A, W), \end{aligned}$$

and

$$\begin{aligned} E\left\{ I(u \le {{\tilde{T}}}_2) \mid Z \right\}&= I(u \le T_1)K_{{{\tilde{C}}},1}( u \mid A, W),\\ E\left\{ I(T_1 \le u< {{\tilde{T}}}_2) \mid Z \right\}&= I(T_1 \le u < T_2)K_{{{\tilde{C}}},2}(u \mid T_1, A, W). \end{aligned}$$

Then

$$\begin{aligned}&E\left\{ \mathop {}\!\textrm{d}M_{{{\tilde{C}}}}^*(u, G_u(Z)) \mid Z \right\} = I(u \le T_1 )K_{{{\tilde{C}}},1}( u \mid A, W) \left\{ \alpha _{{{\tilde{C}}},1}(u \mid A, W)-\alpha ^*_{{{\tilde{C}}},1}(u \mid A, W) \right\} \\&\quad + I(T_1 \le u < T_2)K_{{{\tilde{C}}},2}(u \mid T_1, A, W) \left\{ \alpha _{{{\tilde{C}}}, 2}(u \mid T_1, A, W) \right. \\&\quad \left. - \alpha ^*_{\tilde{C},2}(u \mid T_1, A, W) \right\} , \end{aligned}$$

which is zero when the censoring distribution is correctly specified.

(b)

Note that for any $\int h(u;Z) \mathop {}\!\textrm{d}M^F(u; Z) \in {\mathcal {T}}_i$ for $i=1,2,3$

$$\begin{aligned}&E\left\{ \int h(u;Z) \mathop {}\!\textrm{d}M^F(u; Z) \mid G_r(Z) \right\} - E^*\left\{ \int h(u;Z) \mathop {}\!\textrm{d}M^F(u; Z)\mid G_r(Z)\right\} \\&= \int h(u; Z)\left[ E\left\{ \mathop {}\!\textrm{d}M^F(u;Z) \mid G_r(Z) \right\} - E^*\left\{ \mathop {}\!\textrm{d}M^F(u;Z) \mid G_r(Z)\right\} \right] \end{aligned}$$

Consider first the $1\rightarrow 2$ terms. Note that

$$\begin{aligned}&E^*\left\{ \mathop {}\!\textrm{d}M_{12}(u \mid A, W) \mid G_s(Z) \right\} \\&\quad = I(T_1>s) \bigg [I(u \ge s) \frac{S_1^*(u \mid A, W)}{S_1^*(s\mid A, W)}\left\{ \mathop {}\!\textrm{d}\Lambda _{12}^*(u \mid A, W) \right. \\&\quad \left. - \mathop {}\!\textrm{d}\Lambda _{12}(u \mid A, W) \right\} - I(u< s) \mathop {}\!\textrm{d}\Lambda _{12}(u \mid A, W) \bigg ] \\&\quad + I(T_1 \le s <T_2) \mathop {}\!\textrm{d}M_{12}(u \mid A, W)\\&\quad + I(s > T_2) \mathop {}\!\textrm{d}M_{12}(u \mid A, W), \end{aligned}$$

and

$$\begin{aligned}&E\left\{ \mathop {}\!\textrm{d}M_{12}(u \mid A, W) \mid G_s(Z) \right\} \\&\quad = I(s< T_1) \bigg [- I(u< s) \mathop {}\!\textrm{d}\Lambda _{12}(u \mid A, W) \bigg ] + I(T_1 \le s <T_2) \mathop {}\!\textrm{d}M_{12}(u \mid A, W) \\&\quad + I(s > T_2) \mathop {}\!\textrm{d}M_{12}(u \mid A, W), \end{aligned}$$

so,

$$\begin{aligned}&E\left\{ \mathop {}\!\textrm{d}M_{12}(u \mid A, W) \mid G_s(Z) \right\} - E^*\left\{ \mathop {}\!\textrm{d}M_{12}(u \mid A, W) \mid G_s(Z)\right\} \\&\quad = I(s < T_1)\Bigg [I(u \ge s) \frac{S_1^*(u \mid A, W)}{S_1^*(s\mid A, W)}\left\{ \mathop {}\!\textrm{d}\Lambda _{12}(u \mid A, W) - \mathop {}\!\textrm{d}\Lambda _{12}^*(u \mid A, W)\right\} \Bigg ]. \end{aligned}$$

By similar calculations it holds for the $1 \rightarrow 3$ term that

$$\begin{aligned}&E\left\{ \mathop {}\!\textrm{d}M_{13}(u \mid A, W) \mid G_s(Z) \right\} - E^*\left\{ \mathop {}\!\textrm{d}M_{13}(u \mid A, W) \mid G_s(Z)\right\} \\&\quad = I(s < T_1 \wedge T_2)\Bigg [I(u \ge s) \frac{S_1^*(u \mid A, W)}{S_1^*(s\mid A, W)}\left\{ \mathop {}\!\textrm{d}\Lambda _{13}(u \mid A, W) - \mathop {}\!\textrm{d}\Lambda _{13}^*(u \mid A, W)\right\} \Bigg ]. \end{aligned}$$

and for the $2 \rightarrow 3$ term that

$$\begin{aligned}&E\left\{ \mathop {}\!\textrm{d}M_{23}(u \mid T_1, A, W) \mid G_s(Z) \right\} - E^*\left\{ \mathop {}\!\textrm{d}M_{23}(u \mid T_1, A, W) \mid G_s(Z)\right\} \\&\quad = I(T_1 \le s <T_2)\Bigg [I(u \ge s) \frac{S_2^*(u \mid T_1, A, W)}{S_2^*(s \mid T_1, A, W)}\left\{ \mathop {}\!\textrm{d}\Lambda _{23}(u, u-T_1 \mid A, W) \right. \\&\quad \left. - \mathop {}\!\textrm{d}\Lambda _{23}^*(u, u-T_1 \mid A, W) \right\} \Bigg ]. \end{aligned}$$

## G Simulation details

The following simulation scenarios were considered:

(i) all models are correctly specified: $\zeta =\gamma ^{12}_{AW}=\gamma ^{13}_{AW}=\gamma ^{23}_{AW}= \theta =0$
(ii) censoring and propensity score mis-specified: $\zeta = 2$, $\theta =0.8$ and $\gamma ^{12}_{AW}=\gamma ^{13}_{AW}=\gamma ^{23}_{AW} =0$
(iii) $\Lambda _{12}$ misspecified: $\gamma ^{12}_{AW}=4$ and $\zeta =\gamma ^{13}_{AW}=\gamma ^{23}_{AW}= \theta =0$
(iv) $\Lambda _{13}$ misspecified: $\gamma ^{13}_{AW}=1$ and $\zeta =\gamma ^{12}_{AW}=\gamma ^{23}_{AW}= \theta =0$
(v) $$\Lambda _{23}$$ misspecified: $$\gamma ^{23}_{AW}=1 $$ and $$\zeta =\gamma ^{12}_{AW}=\gamma ^{13}_{AW}= \theta =0$$
(vi) $\Lambda _{12}$ and $\Lambda _{13}$ misspecified: $\gamma ^{12}_{AW}=4$ $\gamma ^{13}_{AW}=1$ and $\zeta =\gamma ^{23}_{AW}= \theta =0$
(vii) $\Lambda _{{{\tilde{C}}}}$ and $\Lambda _{23}$ misspecified: $\theta =0.8$, $\gamma ^{23}_{AW}=1$ and $\zeta =\gamma ^{12}_{AW}=\gamma ^{12}_{AW}=\gamma ^{13}_{AW} =0$
(viii) $\pi$ and $\Lambda _{23}$ misspecified: $\zeta =2$, $\gamma ^{23}_{AW}=1$ and $\gamma ^{12}_{AW}=\gamma ^{12}_{AW}=\gamma ^{13}_{AW} =\theta =0$
